# Host Immune-Metabolic Adaptations Upon Mycobacterial Infections and Associated Co-Morbidities

**DOI:** 10.3389/fimmu.2021.747387

**Published:** 2021-09-23

**Authors:** Alba Llibre, Martin Dedicoat, Julie G. Burel, Caroline Demangel, Matthew K. O’Shea, Claudio Mauro

**Affiliations:** ^1^ Institute of Inflammation and Ageing, College of Medical and Dental Sciences, University of Birmingham, Birmingham, United Kingdom; ^2^ Department of Infectious Diseases, Heartlands Hospital, University Hospitals Birmingham NHS Foundation Trust, Birmingham, United Kingdom; ^3^ Division of Vaccine Discovery, La Jolla Institute for Immunology, La Jolla, CA, United States; ^4^ Immunobiology of Infection Unit, Institut Pasteur, INSERM U1224, Paris, France; ^5^ Institute of Immunology and Immunotherapy, University of Birmingham, Birmingham, United Kingdom

**Keywords:** mycobacteria, macrophage, immunometabolism, host-directed therapies, tuberculosis

## Abstract

Mycobacterial diseases are a major public health challenge. Their causative agents include, in order of impact, members of the *Mycobacterium tuberculosis* complex (causing tuberculosis), *Mycobacterium leprae* (causing leprosy), and non-tuberculous mycobacterial pathogens including *Mycobacterium ulcerans.* Macrophages are mycobacterial targets and they play an essential role in the host immune response to mycobacteria. This review aims to provide a comprehensive understanding of the immune-metabolic adaptations of the macrophage to mycobacterial infections. This metabolic rewiring involves changes in glycolysis and oxidative metabolism, as well as in the use of fatty acids and that of metals such as iron, zinc and copper. The macrophage metabolic adaptations result in changes in intracellular metabolites, which can post-translationally modify proteins including histones, with potential for shaping the epigenetic landscape. This review will also cover how critical tuberculosis co-morbidities such as smoking, diabetes and HIV infection shape host metabolic responses and impact disease outcome. Finally, we will explore how the immune-metabolic knowledge gained in the last decades can be harnessed towards the design of novel diagnostic and therapeutic tools, as well as vaccines.

## Introduction

Mycobacteria have been a major cause of human disease for millennia, with the effects of *Mycobacterium tuberculosis* (*M.tb*) seen in the skeletons of mummified human remains from over 4000 years ago (1). The main mycobacteria of public health importance today are members of the mycobacterium tuberculosis complex (which includes *M.tb, M.bovis, M.africanum, M.microti, M.pinnepedi, M.caprae*), which cause tuberculosis (TB), *Mycobacterium leprae* (*M.leprae*) the cause of leprosy, and nontuberculous mycobacteria (NTM) leading to a wide variety of clinical presentations. NTM include *Mycobacterium ulcerans* (*M.ulcerans*), the cause of Buruli ulcer (BU).

Around 2 billion people worldwide are infected with *M.tb*. Approximately 10 million people fall ill with TB each year and there are around 1.5 million deaths (2). Great progress has been made with TB control over the past decade, but these gains have been undermined to some extent by the ongoing Covid-19 pandemic, especially in less well-resourced settings (3). Current treatment for drug sensitive TB is a minimum of 6 months and considerably longer for drug resistant disease. Shorter more effective treatments are needed, and host-directed therapies (HDT) arise as a promising strategy.

Leprosy caused by chronic infection with *M.leprae* is predominantly a disease of the skin and peripheral nerves. It is curable with a prolonged course of antibiotics. The incidence of leprosy has declined over the past century, but the rate of decline is currently sluggish. A large proportion of endemic countries (118/161) reported new cases in 2019 (202,256 or 26 per million population) (4). Although antibiotics can still cure the disease, permanent changes to nerves can occur leading to lifelong disabilities. There is still a great amount of stigma around the diagnosis of leprosy which can delay detection and effective treatment. Also, weak health systems can make early detection and treatment difficult. Current therapies although effective are prolonged and commonly associated with adverse effects (5).

Both TB and leprosy present a spectrum of clinical manifestations. TB can be described as a dynamic continuum from asymptomatic *M.tb* infection to active infectious disease, including latent infection as well as incipient, subclinical and active TB disease (6, 7). Leprosy also comprises ample clinical variability. The two extremes of the spectrum are tuberculoid and lepromatous forms of the disease, the first being paucibacillary and mild, while the latter is multibacillary and presents increased disease severity (8, 9).

There are over 200 species of NTM identified to date (10), of which the vast majority (over 95%) have not been associated with human disease. NTM are predominantly environmental organisms of low pathogenicity to humans that only cause disease in specific circumstances. An exception is *M.ulcerans*, which causes chronic skin ulcers in immunocompetent individuals, through production of a diffusible cytotoxin called mycolactone (11). BU is, after TB and leprosy, the third most common mycobacterial disease worldwide and together with leprosy one of the 20 Neglected Tropical Diseases prioritized by the WHO (12). West African countries are the worst impacted by BU, with prevalence rates reaching 26.9 cases per 10,000 in Benin. Other NTMs primarily affect immunocompromised people. Recently, NTM infections have been associated with health care procedures with infections due to *M.chimerae* occurring after cardiac surgery (13) and infections with rapid growing NTM’s such as *M.abscessus* being associated with cosmetic surgery procedures (14). *M.abscessus* is also associated with progressive lung infection in patients with cystic fibrosis (15). Treatment of NTM infections is complex requiring multiple prolonged antibiotics which may not be effective or well tolerated. Overall, novel treatments for mycobacterial diseases are needed. HTDs are a promising approach but we need a greater understanding of how the host responds to infection.

## Macrophages as Targets of Mycobacterial Infection

Macrophages not only play an essential role in the host immune response to mycobacteria, but they also are mycobacterial targets. They fulfil a variety of essential functions in homeostasis and disease, including phagocytosis, uptake and killing of pathogens, tissue repair and inflammation resolution (16, 17). Mirroring their wide range of functions, macrophages constitute a highly diverse and heterogeneous population (18, 19). Traditionally, they have been classified into M1, i.e. classically-activated (LPS + IFNγ) and pro-inflammatory, and M2, i.e. alternatively-activated (IL-4) and anti-inflammatory. While the oversimplified M1/M2 dichotomy has been a useful tool when studying macrophage diversity, most macrophages exist as a spectrum and contain features of both (20, 21). Additionally, it is increasingly recognised that specific environmental cues can promote certain macrophage phenotypes and functions, demonstrating their plasticity. Metabolically, M1-like macrophages are predominantly glycolytic and present a broken TCA cycle (22). Their pentose phosphate pathway as well as fatty acid synthesis are highly active, ensuring availability of biosynthetic precursors. In contrast, M2-like macrophages mainly rely on oxidative metabolism, present an intact TCA cycle and fatty acid oxidation is upregulated. This review will explore macrophage metabolic diversity and flexibility and how these metabolic changes can drive specific phenotypes and functions, focusing on their relevance in the context of mycobacterial pathogenesis.

*M.tb* infection occurs when aerosols containing the bacilli are inhaled by a susceptible host. When *M.tb* reaches the lung, alveolar macrophages (AM), the first cellular target (23), are infected. Infected AMs can migrate to the lung interstitium, facilitating infection of other cell types, including newly-recruited monocyte-derived macrophages and neutrophils. Although abundant evidence points towards *M.leprae* being transmitted through the respiratory route (24, 25), the precise mechanisms for *M.leprae* spread remain to be fully elucidated (26). *M.leprae* primary cell targets are macrophages and Schwann cells, the latter contributing to build the myelin sheath that covers nerve fibres. Following introduction into the skin, *M. ulcerans* bacilli are phagocytosed by macrophages and multiply intracellularly until bacterial production of mycolactone causes host cell apoptosis (11, 27). Therefore, macrophages are a clear target for mycobacterial pathogens which have evolved successful strategies to survive and replicate within them (28), including metabolic manipulation.

In the context of mycobacterial disease, macrophages play an essential role in driving innate and adaptive immune responses and inflammation, while mediating both tissue destruction and repair (29). Two major macrophage populations cohabit in the lung: AMs and interstitial macrophages (IMs). They are distinct at the ontogenic, phenotypic, metabolic and functional levels (30). AMs display an M2-like phenotype (31) with predominant oxidative metabolism (30, 32), similar to IL-4 treated hMDM (33). In contrast, IMs are mainly derived from recruited monocytes (34, 35), and this has been particularly shown in the context of *M.tb* infection (30, 36). IMs are M1-like and operate at high levels of glycolysis (30). Other types of macrophages relevant in the context of mycobacterial infection include epithelioid cells, multinucleate giant cells (MGCs) and foamy, lipid-rich macrophages (37). MGCs are the result of macrophage fusion within the granuloma, organised cellular aggregates hallmark of TB and leprosy (38).

In leprosy, skin lesions from the milder, tuberculoid forms of the disease have been shown to have an M1-like macrophage predominant population, whereas in more severe skin lesions of multibacillary patients the balance shifts towards M2-like macrophages (39).

BU manifests as chronic ulcerative skin lesions with a distinctive lack of pain and inflammation, which if untreated enlarge over time (40). Tissue necrosis, local analgesia and defective inflammation are all attributed to bacterial production of mycolactone, a diffusible macrolide targeting the entry point of the secretory pathway in eukaryotic cells: the Sec61 translocon (11, 41, 42). By inhibiting Sec61, mycolactone prevents host cell’s production of secreted proteins, and most of its transmembrane proteins, leading to their cytosolic degradation by the proteasome (43–45). In the skin regions surrounding bacterial foci, complete and sustained inhibition of Sec61 in host cells triggers endoplasmic reticulum (ER) stress responses culminating in apoptosis (46). In immune cells recruited to the site of infection, including macrophages, exposure to non-cytotoxic concentrations of mycolactone prevents the production of cytokines, chemokines and the transduction of receptor-mediated signals, thereby the generation of protective immune responses (41). How *M.tb*, *M.leprae* and *M.ulcerans* impact the metabolic reprogramming of immune cells is explored in the following section, and is summarised in **Table 1**.

**Table 1:** M1-like vs M2-like macrophages in mycobacterial infection.

	M1-like	M2-like
**Baseline**	Predominantly glycolytic Pro-inflammatory Microbicidal Key markers: CD86, iNOS, ROS	Mainly reliant on oxidative metabolism Anti-inflammatory Tissue repair Key markers: CD206, CD163, Arg
***M.tb***	Interstitial macrophages Glycolysis needed for *M.tb* control (47) *M.tb* restricts glycolysis (48–50)	Alveolar macrophages More permissive to *M.tb* growth (30)
***M.leprae***	Predominant tuberculoid, lesions (39, 51) Blocks M1 polarization in infected monocytes (52)	Predominant in severe lesions (39, 51) Favor bacterial persistence (53) Infections promote M2 phenotype in hMDMs (54, 55) Promotes Treg phenotype (54, 55)

Summary of the key distinctive factors between the M1-like and M2-like macrophage populations. Each population plays a different role in the context of M.tb and M.leprae infection, and have specific capabilities to combat infection, resulting in differential outcome.

## Immune-Metabolic Adaptations of the Macrophage to Mycobacterial Infection

**Figure 1:**
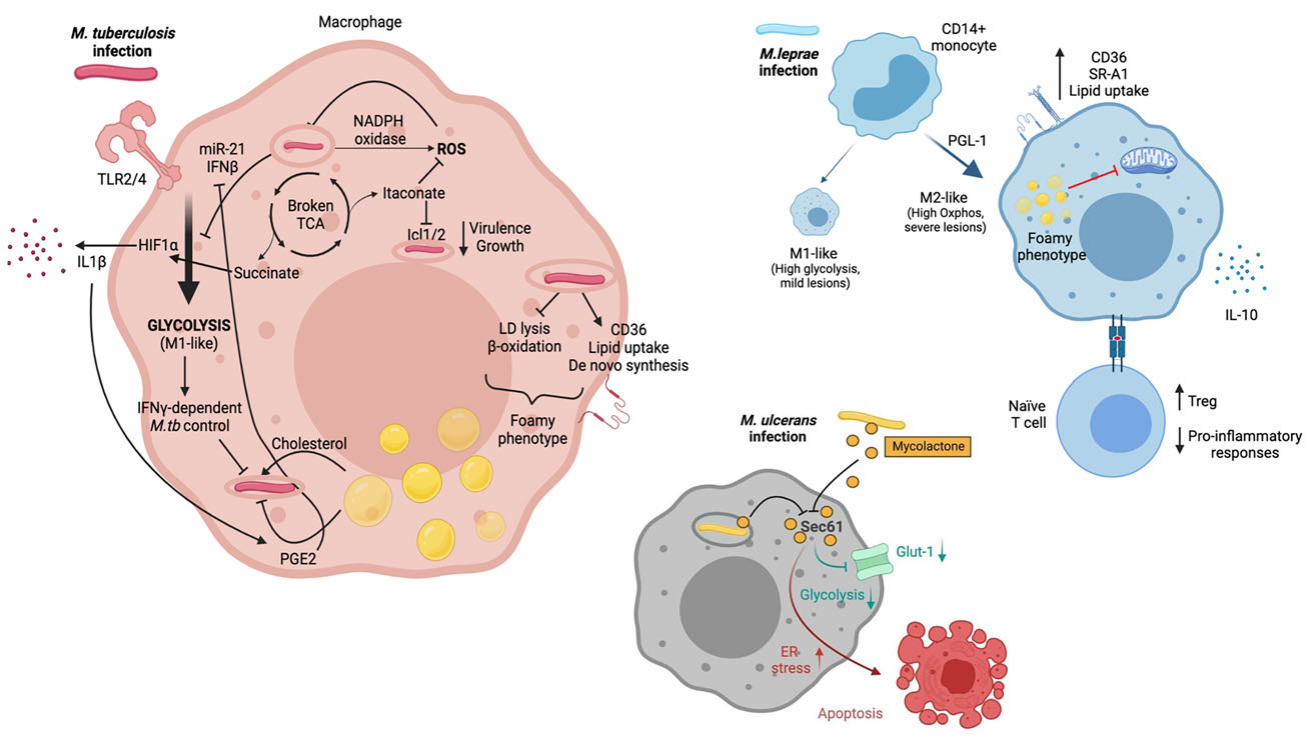
*M.tb*, *M.leprae* and *M.ulcerans* infect macrophages and alter their metabolism. *M.tb* is primarily sensed by host macrophages through TLR2 and TLR4. Macrophage metabolism shifts towards glycolysis (M1-like), which allows infection control. *M.tb* has developed strategies to counteract this metabolic switch though induction of miR-21 and IFNβ. M1-like macrophages present a broken TCA cycle, with elevated succinate concentrations that stabilise HIF1α, essential for induction of glycolysis and secretion of pro-inflammatory cytokines such as IL-1β. Itaconate also arises as a consequence of a broken TCA cycle and has multiple immune-regulatory functions, including inhibition of isocitrate lyases Icl1/2*. M.tb* infection triggers ROS production through NAPDH oxidase. It also promotes increased intracellular lipid content. This results in the development of lipid droplets and the foamy macrophage phenotype, which can be beneficial (i.e. providing cholesterol as nutrient) or detrimental (i.e. secretion of prostaglandins) for the pathogen. *M.leprae* infection of CD14+ monocytes drives macrophages towards an M2-like phenotype, with a key role for PGL-1. M2-like macrophages rely on oxidative metabolism and are associated with severe lesions. Upon *M.leprae* infection, macrophages upregulate CD36 and SR-A1 which translates in increased lipid uptake. Lipid droplets and the foamy phenotype have been linked to suppressed mitochondrial function. Furthermore, *M.leprae*-infected macrophages promote a Treg phenotype when interacting with naïve T cells, together with an impairment of pro-inflammatory cytokine release. Mycolactone released by phagocytozed and extracellular *M. ulcerans* bacilli diffuses into the cytoplasm of host macrophages and neighboring cells, respectively, gains access to the Sec61 translocon and blocks its activity. An immediate effect of Sec61 blockade by mycolactone is the downregulation of secretory and transmembrane proteins, among which the glucose importer Glut-1, likely resulting in decreased glycolysis. Sustained Sec61 blockade in mycolactone-exposed cells, including macrophages, triggers ER stress responses culminating in apoptosis. *Created with BioRender.com*.

This section provides an overview of the current knowledge regarding the effects of *M.tb*, *M.leprae* and *M.ulcerans* on host macrophage glycolysis, oxidative and lipid metabolism (**Figure 1**).

### Glycolysis Versus Oxidative Metabolism: Shaping Macrophage Polarisation

Activation of Toll-like receptors (TLRs) by mycobacterial components induces dynamic and coordinated changes in the energy metabolism of host macrophages similar to those occurring during macrophagic differentiation into the M1-like phenotype. They include a switch towards glycolysis, a disruption of the TCA cycle leading to the accumulation of succinate, and an impaired oxidative phosphorylation. Such alterations are promoted by the Hypoxia-Inducible Factor (HIF)-1α, an oxygen sensor and key glycolysis regulator that is activated by the TCA cycle intermediate succinate and mediates the production of Interleukin (IL)-1β (56). Induction of aerobic glycolysis and HIF-1α are beneficial for both innate and IFNγ-dependent control of intracellular *M.tb* infection by host macrophages (47, 57, 58). Importantly, in contrast to killed *M.tb* and the vaccine strain Bacille Calmette-Guérin (BCG), live *M.tb* was recently shown to specifically prevent the glycolytic switch in infected macrophages, pointing towards *M.tb* having evolved specific strategies to modulate host cell metabolism to its own benefit (48–50). Thus, there is an arms race to control macrophage metabolism as it is essential in determining infection outcome, and this topic has been extensively covered in recent excellent reviews (59–61). Validating this idea, it was shown in a mouse model of *M.tb* infection that ontologically and metabolically distinct lung macrophage populations (AMs and IMs) have differential capacities to control bacterial burden (30). Specifically, IMs which are predominantly monocyte-derived and glycolytic, present an increased ability to control *M.tb* growth compared to AMs, a subset of embryonic origin committed to fatty acid oxidation. Although these findings require further validation in humans, a recent single cell RNA-seq study demonstrated that the majority of lung macrophage populations are conserved between mouse and human (62).

Reprogramming of energy metabolism in *M.tb*-infected macrophages is also associated with increased levels of NADPH oxidase and inducible nitric oxide synthase (iNOS), promoting the production of reactive oxygen species (ROS) including nitric oxide (NO) with antimycobacterial activity (63, 64). In addition to yielding succinate, disruption of the TCA cycle upon *M.tb* infection promotes the generation of itaconate from cis-aconitate by aconitate decarboxylase, also known as immune-responsive-gene 1 (IRG1). Although itaconate suppresses the production of inflammatory cytokines and ROS by infected macrophages (65, 66), it directly inhibits *M.tb* enzymes isocitrate lyases Icl1/2, which are required for bacterial virulence and growth *in vivo* (67). IRG1 expression was shown to potentiate macrophage capacity to control intracellular *M.tb* (68) and prevent immunopathology in a mouse model of *M.tb* infection (69), demonstrating the key importance of this metabolic pathway in host defence against *M.tb*.

Studies in Schwann cells suggested that infection with *M.leprae* may also triggers major metabolic reprogramming in infected macrophages. Following infection by *M.leprae*, Schwann cells increase the expression of insulin-like growth factor (IGF), upregulating glucose transporter 1 (GLUT-1) and glucose uptake by Akt signalling (70). Glucose is redirected from glycolysis to the pentose phosphate pathway through the activation of G6PD, increasing the carbon flux to lipid biosynthesis, while both mitochondrial activity and lactate production are reduced (71, 72). Since *M.leprae* infection success depends on the pentose phosphate pathway, which generates reducing power for glutathione antioxidant system, it was proposed that *M.leprae* subverts host cell glucose metabolism to facilitate glutathione regeneration and thereby free-radical control (71).

*In vitro* experiments demonstrated that M1-like macrophages acquire M2-like phenotypes in the presence of *M.leprae* and apoptotic cells (which occur in skin lesions), contributing to mycobacterial persistence (52). Exposure of human CD14+ monocytes to *M.leprae* (MOI 5:1) inhibited M1 polarization, and this effect was likely mediated by the lipid component PGL-1 (73). IL-10 (anti-inflammatory) and IL-15 (pro-inflammatory) were shown to drive distinct macrophage responses which translated into progressive versus self-healing leprosy lesions, respectively (54). Live (but not killed) *M.leprae* promoted an M2-like phenotype in hMDM, based on IL-1β, IL-6, TNF, IL-10, CD163 and MHC-II expression (55). This M2-skewing was confirmed by measuring IL-10 and IL-12 transcript and protein levels in a different study (74). When *M.leprae*-infected macrophages were exposed to naïve T cells, diminished pro-inflammatory responses together with increased T regulatory phenotypes were reported (55, 74). These changes occurred using *M.leprae* harvested from both tuberculoid and lepromatous skin lesions (74). These findings suggest that the M2-like macrophages with predominant oxidative metabolism – same as in the context of *M.tb* infection - would favour pathogen persistence rather than infection resolution.

Production of mycolactone by intracellular *M.ulcerans* causes the apoptosis of host macrophages, and bacteria grow primarily extracellularly in infected skin during active BU (11). However, mycolactone released by bacteria diffuses broadly in infected organisms, interfering with the metabolism of both skin-resident and more distant cells (11, 75–77). Mycolactone-mediated Sec61 blockade is likely to impair host cell energy metabolism *via* the downregulation of nutrient tranporters, such as the glucose transporter Glut1 (SLC2A1). In support of this hypothesis, our metabolomic analysis of Jurkat T cells exposed to mycolactone revealed decreased intracellular levels of Glut1 substrates, glucose-1-phosphate and mannose-6-phosphate, suggesting impaired glycolytic activity (77). We speculate that during infection with *M.ulcerans*, inhibition of Sec61 impairs glycolysis reprogramming in infected macrophages, and more generally in all mycolactone-exposed immune cells.

### Lipid Metabolism

In addition to reprogramming energetic metabolism, *M.tb* infection rewires the lipid metabolism of host macrophages through inhibition of catabolic pathways (lipid droplet [LD] lysis and β-oxidation of fatty acids) and concomitant activation of lipid uptake, mobilization and *de novo* synthesis (78). This leads *M.tb*-infected macrophages to acquire a foamy phenotype, due to the cytoplasmic accumulation of LDs mainly composed of triacylglycerol (TAGs), a storage form of fatty acids, and cholesteryl esters. Whether LD accumulation is beneficial to host macrophages or intracellular *M.tb* is a matter of debate. Since intracellular *M.tb* has the ability to import fatty acids deriving from host TAGs and foamy macrophages are a hallmark of chronic TB, accumulation of LDs in infected macrophages may provide the pathogen with essential nutrients (37, 79–81). However, recent data indicate that LD maintenance requires IFNγ-driven induction of HIF-1α, which inhibits lipolysis and prevents *M.tb*’s acquisition of host lipids (82). Besides, LDs are the major sites of eicosanoid production including the anti-mycobacterial prostaglandin E2 (PGE2) (83), and have direct antibacterial properties (84). PGE2 is required for *M.tb* control (85, 86) Leukotriene B4 (LTB4) is elevated in pulmonary TB compared to latent *M.tb* infected individuals (LTBI) (87) and contributes to *M.tb* immunopathogenesis (86). Beside eicosanoids, fatty acids released by LD lysis can be shuttled across the mitochondrial cell wall for β-oxidation, generating acetyl-CoA that enters the TCA cycle, and co-enzymes used in the respiratory chain to produce ATP. Interestingly, inhibiting fatty acid oxidation augmented macrophage ability to control *M.tb* infection (30, 53). Rather than starving intracellular *M.tb* from lipid nutrients, blocking β-oxidation of fatty acids may favour the generation of mitochondrial ROS that promote the phagosomal recruitment of NADPH oxidase and the xenophagic elimination of *M.tb* (53).

Similar to *M.tb*, *M.leprae* upregulates lipid uptake and biosynthetic pathways in infected macrophages, particularly cholesterol (88, 89). Mechanistically, infection with *M.leprae* increases macrophage expression of scavenger receptor (SR)A-I and CD36, promoting the foamy macrophage phenotype (90, 91). The mycobacteria seem to take shelter within lipid bodies, formed abundantly by host cells, possibly as a strategy to cover and hide surface antigens from innate immune receptors in the cytosol (92, 93). Contrary to *M.tb* (94), *M.leprae* cannot degrade or utilize cholesterol as a nutritional source (92), leaving the mechanism by which host cholesterol metabolism supports its *in vivo* persistence undefined. Notably, *M.leprae* infection reduces host cell mitochondrial activity in a distinctive manner (88). Oliveira et al. proposed that cytosolic accumulation of lipids in *M.leprae*-infected macrophages may contribute to mitochondrial shutdown and suppression of their innate immune functions (88).

Besides, macrophages infected with *M.tb* deprive intracellular bacteria from essential micronutrients like iron and manganese, while using copper and zinc to poison them (95). In turn, *M.tb* has developed sophisticated strategies to ensure micronutrient acquisition and resist metal toxicity. Interestingly, iron release prevails in AMs facilitating *M.tb* access to iron, whereas IMs have the capacity to sequester it, contributing to an iron starvation *M.tb* phenotype (96). Further information on this topic can be found in an excellent recent review by Neyrolles et al. (95). Proteins involved in iron uptake and metabolism are upregulated in lepromatous leprosy lesions, compared to tuberculoid forms, suggesting an association between iron storage in *M.leprae*-infected macrophages and intracellular bacterial persistence (97).

Together, these studies revealed aerobic glycolysis and fatty acid oxidation as key metabolic pathways enhancing or decreasing the anti-mycobacterial responses of macrophages, respectively. They highlighted species-specific mechanisms used by *M.tb*, *M.leprae* and *M. ulcerans* to subvert the glycolytic switch that is induced by TLR stimulation in infected macrophages, and take advantage of the increased lipid anabolism in host macrophages.

## Metabolic-Epigenetic Crosstalk in Mycobacterial-Infected Macrophages

The macrophage metabolic adaptations to mycobacterial infection translate into changes of the intracellular metabolome. The concentration of particular metabolites therefore increases with the predominance of certain metabolic pathways. For example, lactate generation is enhanced with glycolysis, and so are citrate and acetyl-CoA with an active TCA cycle. These metabolic intermediates have the potential to post-translationally modify a wide range of proteins (e.g. histones), increasing proteome diversity and modulating function, including immune function, according to the cell needs (98–100). DNA and histones can thus be modified through metabolic substrates (e.g. methionine, acetyl-CoA, lactate), and metabolic genes can also be targets of such modifications (101). There is increasing recognition that epigenetics play an important role in shaping host-pathogen interactions and infection outcomes.

Zhang and colleagues showed that macrophage histones can be lactylated promoting an M2-like phenotype upon bacterial challenge (102), although this idea has been recently challenged (103). Since elevated glycolysis and lactate production occur in the lung of *M.tb*-infected hosts [reviewed in (104)], lactate could potentially drive macrophages towards an M2-phenotpye, favouring *M.tb* survival.

*M.tb* components switch host cellular metabolism toward aerobic glycolysis in human peripheral blood mononuclear cells (PBMC) through a TLR2-dependent but NOD2-independent mechanism which is partly mediated *via* the activation of the AKT/mTOR pathway (58). This seems to be of functional relevance as inhibition of the AKT/mTOR pathway inhibits cellular responses to *M.tb* both in human PBMC and a murine model of TB (58). Insights into the possible mechanisms underlying this relationship has come from studies of BCG vaccination, which has been shown to be a potent inducer of trained immunity due to cellular metabolism reprogramming arising from epigenetic changes (105). BCG-stimulated monocytes have been shown to undergo chromatin remodeling due to histone modification, namely increases in H3K4me3 and H3K9me3 at promoter sites of essential glycolytic genes. The resultant activation of the AKT/mTOR/HIF-1α pathway switches cellular metabolism from oxidative phosphorylation to aerobic glycolysis and as a consequence facilitates increased production of cytokines such as TNF and IL-6 that promote mycobacterial killing (106, 107). Conversely, these epigenetic changes are dependent on the induction of metabolic processes, as inhibition of glycolysis results in reversal of changes in H3K4me3 and H3K9me3 at promoter sites of TNF and IL-6 (107). In contrast to BCG, *M.tb* has been shown to impair macrophage trained immunity through activation of the type I interferon/iron axis in hematopoietic stem cells (108). This immune-metabolic reprogramming also resulted in suppressed myelopoiesis, overall enhancing host susceptibility to *M.tb* infection (108).

Another recently described epigenetic modification shown to cause immunomodulation in *M.tb* infection involves Alu repeat elements. Alu repeats are mobile interspersed repetitive DNA sequences that are transposable from one site in the genome to another, resulting in mutations, insertions and recombination events in protein-coding mRNAs (109). Analysis of genes adjacent to H3K4me1-associated Alu repeats linked to macrophage metabolic responses to *M.tb* infection has shown that Liver X Receptor-α signaling can be initiated at response elements present in Alu repeats and significantly reduces *M.tb* viability by altering cholesterol metabolism and enhancing macrophage apoptosis (110). Furthermore, levels of *Alu* methylation have been found to be significantly lower in paediatric TB patients and the detection of *Alu* DNA methylation may serve as a diagnostic and prognostic tool of TB disease in this population (111).

## Co-Morbidities Impact on Metabolism and Immunity

**Figure 2:**
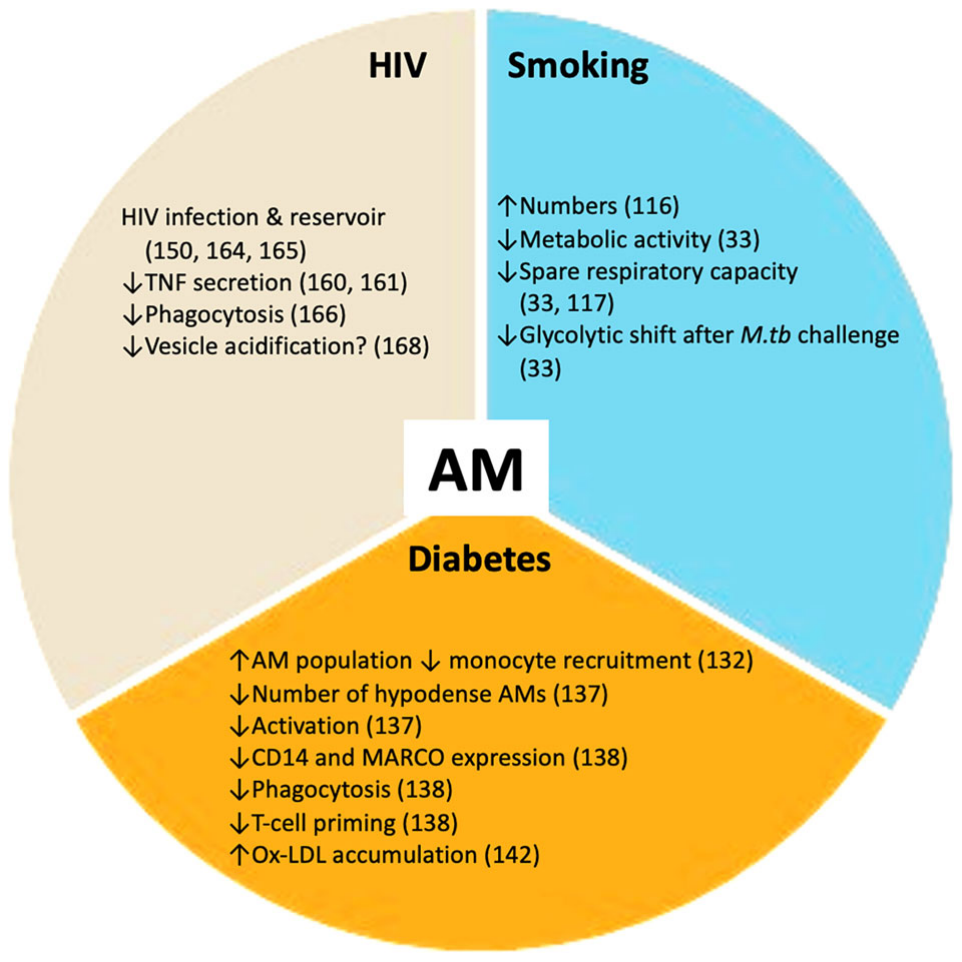
TB co-morbidities impact alveolar macrophages (AMs). Smoking, HIV infection and diabetes mellitus perturb the healthy alveolar macrophage phenotype. These co-morbidities promote changes in alveolar macrophage metabolism and essential functions including phagocytosis and cytokine secretion. These disturbances render the alveolar macrophage population more susceptible to *M.tb* infection. ↑ increased; ↓ decreased.

Vulnerability to infection with *M.tb* and progression to active disease can be affected by several co-morbidities and social risk factors. The main co-morbidities associated with TB progression and poor outcomes are HIV infection, diabetes, renal disease and smoking. Social risk factors such as excess alcohol consumption, air pollution, incarceration and poor housing are also important. Diabetes, tobacco smoking and HIV infection have a profound impact on the host metabolic state, at both the macrophage (**Figure 2**) and systemic levels. Understanding how these metabolic changes occur will contribute to elucidating why these particular conditions worsen TB.

### Smoking

The fact that tobacco smoking impacts the immune system and increases the prevalence of both respiratory and distal organ-associated diseases has been known for decades [reviewed in (112–116)]. Cigarette smoke (CS) contains abundant compounds (including toxins and carcinogens) which can directly modify immune function [reviewed in (115)].

Few studies have investigated the direct metabolic impact of smoking in *M.tb* infection. A macrophage shift to glycolysis is essential for effective control of *M.tb* (30, 47, 49). AMs from smokers presented decreased capacity to control H37Ra *M.tb* compared to non-smokers (117). This was probably due to impaired secretion of key cytokines for infection control, including IL-1β, TNF and IFNγ, and some of these altered immune responses remained in AMs from ex-smokers (117).

Both reduced metabolic activity (measured by oxygen consumption rate [OCR] and extracellular acidification rate [ECAR]) and metabolic reserves (glycolytic reserve and spare respiratory capacity [SRC]) have been reported at baseline in AM from smokers, measured by extracellular flux analysis (33). Interestingly, baseline metabolism of smokers’ AMs was skewed towards glycolysis, matching previous results (118). AMs from smokers showed diminished glycolysis than AMs from non-smokers when challenged with *M.tb*, measured by extracellular flux analysis and lactate secretion (33). No differences in oxidative metabolism were detected. Of note, the essential shift towards glycolysis (ECAR/OCR) for *M.tb* control was severely impaired in smokers’ AMs compared to their non-smokers counterparts, and was confirmed at the transcriptional level (2-fold change reduction in the glycolysis rate-limiting enzyme hexokinase 1). This translated in a trend towards attenuated pro-inflammatory responses (IL-1β and PGE2 secretion). The described deficient shift to glycolysis, decreased lactate and IL-1β secretion were confirmed in an *in vitro* model of hMDMs treated with CSE (33). The bioenergetic profiling experiments were performed using H37Rv γ-irradiated *M.tb*. This is important since live/dead *M.tb* have been shown to induce profoundly different metabolic adaptations in hMDMs measured by extracellular flux analysis (48). Other studies using hMDM and a THP-1 model and CSE have shown impaired responses to LPS, based on reduced basal and induced glycolysis, NLRP3 activation and subsequent IL-1β and IL-18 secretion (119).

Although further mechanistic studies are needed, there is enough evidence supporting the notion that CS causes dysfunctional AM metabolic profiles, which directly impact the orchestration of effective immune responses against respiratory pathogens, and might help explaining the worsening of TB reported in the smoking population.

### Diabetes Mellitus

#### The Increasing Overlap of Two Epidemics

Up until a few years ago, the epidemics of TB and diabetes mellitus (DM) were, for the most part, geographically disconnected. Today, the geographic overlap between these two epidemics arises as a worldwide threat, as TB and diabetes have the potential to make each other worse (120, 121). It is estimated that about 15% of current TB cases are associated with T2D (122, 123). Here, we will discuss how diabetes detrimentally impacts TB.

Animal studies have further confirmed that DM worsens TB outcomes. In a mouse model of DM using streptozotocin (STZ) to destroy pancreatic islets, macrophages presented a 90% reduction in their phagocytic capacity, although their intracellular killing abilities were intact (124). When these mice were challenged with *M.tb* (Schacht strain) 90% of them died, compared to 10% in the non-diabetic group (124). Other mice, rat and guinea pig models of diabetes/hyperglycaemia have reported increased bacterial burden compared to control animals, together with diminished IFNγ responses (125–129).

#### Immune Alterations

Chemotaxis of monocytes has been shown to be impaired in diabetic patients (130, 131), potentially affecting recruitment into the lung. Furthermore, monocytes from DM patients presented diminished binding and phagocytic capacity towards H37Rv *M.tb* compared to healthy controls (132).

Within the lungs of diabetic C57BL/6 mice (STZ model) and using an aerosol challenge model of Erdman *M.tb*, it was shown that the increased TB susceptibility may arise from delayed innate immune responses (133). In particular, at 2 weeks post-infection (pi) there was abundant recruited monocytes at the site of infection in the control group. In contrast, in the lungs of diabetic mice, *M.tb*-infected AMs prevailed, and monocyte recruitment was limited (133). This translated in delayed delivery of *M.tb*-antigens to the lymph nodes, and delayed presence of IFNγ+ *M.tb*-specific T cells in the lymph nodes and in the lungs compared to the non-diabetic group, slowing appearance of effective immune responses and potentially contributing to bacterial persistence (133). The mechanisms underlying impaired chemotaxis/recruitment remain unknown. The reliance of AMs on oxidative metabolism which facilitates *M.tb* persistence, in comparison to the more glycolytic recruited monocytes, could further explain the observed differences in mycobacterial control.

PBMCs from healthy individuals treated with *M.tb* lysate in high concentrations of glucose (40mM, but not 25mM or lower) resulted in increased secretion of TNF, IL-1β, IL-6, but not IFNγ IL-17A and IL-22 (134). Differentiated macrophages at 25 mM glucose promoted enhanced cytokine production after stimulation with *M.tb* lysate and LPS. However, no differences were reported in phagocytosis and *M.tb* killing capacities (134). Macrophages differentiated from healthy individuals and diabetic patients were characterised at baseline and after *M.tb* infection (MOI 5:1 for 24h) with different strains (135). Expression of key molecules for antigen presentation (HLA-DR, CD80 and CD86), the inhibitory molecule PD-L1, and cytokines/chemokines secretion patterns differed between DM patients and controls. MDMs from T2D patients presented attenuated capacity to bind, internalise and clear *M.tb*, and worse outcomes were reported with more virulent strains (135). Similarly, MDMs from chronic diabetic patients presented impaired *M.tb* killing capacity (136). Monocytes from T2D patients and healthy controls infected with *M.tb* presented similar bacterial growth (137). However, monocytes cultured at 30mM glucose to mimic hyperglycaemic conditions, compared to monocytes cultured at 11mM glucose, presented decreased IL-8 production (a key neutrophil chemoattractant) as well as increased H37ra *M.tb* survival 3 days pi (137).

In the particular context of AM, TB-DM patients presented decreased numbers of a particular hypodense AM subset, compared to TB only patients (138). Within the active pulmonary TB patients, a negative correlation was observed between the percentage of hypodense AMs and sputum bacterial load, together with disease severity assessed by chest X-ray. Overall AMs from TB-DM patients were less activated (138). Although primarily reporting descriptive findings, this is, to our knowledge, the first study showing direct impact of DM in the AM lung-resident population. Another key study focusing on AMs used the STZ mice diabetes model and showed that AMs from diabetic mice presented reduced CD14 and MARCO expression, the latter being essential in the recognition of trehalose 6,6′-dimycolate (TDM) within the bacterial cell wall (139). This translated in reduced *M.tb* (Erdman strain) phagocytosis and was specific to the AM population (peritoneal or BMDM did not present this altered phenotype). These AMs defects resulted in impaired T-cell priming, which was observed when AMs from diabetic animals were transferred to control animals (139).

Other major immune cell types including dendritic cells, neutrophils, natural killer cells and T cells are also impacted in the context of diabetes and hyperglycaemia. It is beyond the scope of this review to explore their precise mechanisms, and they have been reviewed elsewhere (140, 141).

#### Immune-Metabolic Alterations Beyond Hyperglycaemia

DM not only comprises hyperglycaemia but also a wide range of further metabolic alterations [reviewed in (142)] which have the potential to impact immune responses to *M.tb* (i.e. dyslipidaemia, redox and hormone balance). DM is often associated with dyslipidaemia (e.g. increased oxidised-low density lipoproteins [ox-LDLs)] and although it is difficult to establish causal links, it is well described that *M.tb* thrives in lipid-rich environments. Ox-LDLs accumulate in AMs of guinea pigs infected with *M.tb*, increasing bacterial burden (143). Similarly, *in vitro* human studies showed that ox-LDLs promoted *M.tb* survival by impairing lysosomal function (144).

It is challenging to draw conclusive answers regarding the underlying mechanisms of TB enhanced susceptibility in DM patients due to the limited number of human studies and the variety of models used (ie. mouse and human monocytes/macrophages, from diabetic subjects or treated with varying concentrations of glucose). Nonetheless, there is ample evidence suggesting altered function of the myeloid compartment in the context of DM, including chemotaxis, bacterial recognition, phagocytosis, cytokine secretion and metabolism, which could facilitate TB disease. The described immune alterations have the potential to impact the outcome of other infectious diseases [reviewed in (145, 146)]. Although there is scarce literature exploring the link between diabetes and leprosy, early studies reported an increase in diabetes in lepromatous leprosy patients (147).

### HIV

According to the WHO, people living with HIV have an increased risk (16-27 times) of developing active TB compared to non-infected individuals (2). One of the key mechanisms behind this enhanced risk is the fact that HIV causes CD4+ T cell depletion [reviewed in (148)], a cell subset essential for control of *M.tb* infection (149–151). There is increasing awareness of HIV-driven impairment in the innate immune compartment as a key contributor to increased TB risk in *M.tb*-infected individuals. Here, we explore the impact of HIV infection on macrophages and how that may facilitate TB progression.

Monocyte chemotaxis (152) and monocyte oxidative burst capacities have been shown to be impaired in HIV-infected individuals (153). HIV infection downregulated essential TLRs in the myeloid compartment which are essential for *M.tb* recognition (154, 155). *In vitro*, HIV infection of hMDMs promotes an M1-like phenotype (156) and impacts macrophage metabolism, inducing mitochondrial fusion, reduced oxidative phosphorylation and no changes in glycolysis (157, 158). Enhanced lipid accumulation together with increased uptake of ox-LDL was reported in hMDMs from HIV-infected individuals compared to controls, which could potentially contribute to the foamy macrophage phenotype and aid *M.tb* survival (159). HIV-infected hMDMs prevented GM-CSF-mediated activation of STAT5A, a signalling pathway essential for TB control (160). TNF secretion was reduced in HIV/*M.tb* co-infected macrophages compared to *M.tb* alone in a THP-1 cell model and human AMs (161, 162), as well as TNF-dependent apoptosis (161–163). There is evidence of HIV capacity to infect AMs (164, 165) which can act as viral reservoirs (166). HIV-infected AMs present impaired phagocytic function (167), and HIV inhibits phagocytosis in hMDMs in a Nef-dependent manner (168). Restricted vesicle acidification in AMs from HIV-TB patients has been reported (169). One study in HIV-TB coinfected individuals reported no differences in phagocytosis or acidification capacities due to HIV infection (170). The overall evidence suggests that HIV makes AMs more permissive to *M.tb* and promotes early bacterial growth, but further research is needed to precisely elucidate the role of macrophage metabolism in this co-infection setting.

In contrast to TB, an increased risk of leprosy or enhanced severe disease has not been described in HIV-infected individuals [reviewed in (171)]. However, leprosy patients receiving highly active antiretroviral therapy (HAART) are at increased risk of reversal reaction (RR), an inflammatory exacerbation (172). The monocyte/macrophage phenotype has been shown to be distinct in skin lesions from RR/HIV patients compared to RR alone (173) including increased expression of CD209, vascular endothelial growth factor (VEGF), arginase 2 (ARG2) and PPARγ in the former. The clinical implications of these findings remain unknown. HIV-BU co-infection is rare and there is very limited research on the topic. However, current studies point towards BU patients being more likely to be infected with HIV, and HIV infection increasing BU severity (174, 175).

## Identifying Immune-Metabolic Host Therapeutic Targets

**Figure 3:**
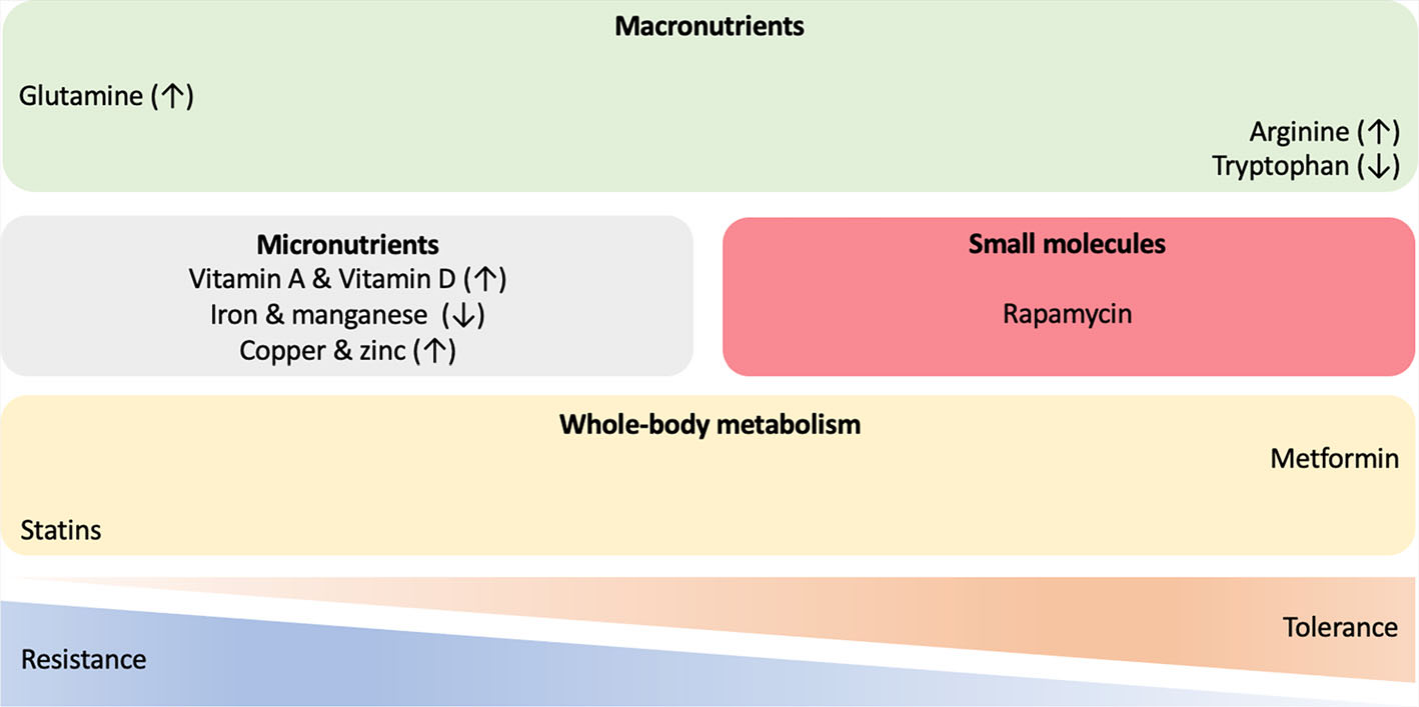
Host-directed therapies in mycobacterial infections. A wide range of approaches and molecules are being investigated to identify novel immune-metabolic targets for treatment of mycobacterial diseases. Therapeutic interventions with macronutrients, micronutrients and small chemical molecules, as well as compounds targeting whole-body metabolism have the potential to shift the resistance/tolerance balance of the host when challenged with mycobacteria. ↑ increased; ↓ decreased.

Given the significant disease burden of TB and leprosy, the rise in drug resistance and the challenges associated with the development of novel anti-mycobacterial agents, HDT strategies which augment host responses to mycobacterial infections, have become an increasing focus of interest (176–178) (**Figure 3**). Greater understanding of the role of immunometabolism in both protection against, and susceptibility to, infection provides potential targets to promote resistance and modulate tolerance to chronic infection.

As discussed, it is well established epidemiologically that individuals with metabolic conditions such as T2D are at increased risk of developing active TB disease, suggesting not only the important role of host metabolic regulation in anti-*M.tb* responses but also pathways for possible therapeutic intervention (179).

In the context of T2D, the antihyperglycemic biguanide drug metformin appears to be a good candidate to modulate mycobacterial tolerance [reviewed in (180)]. Among patients with T2D metformin reduces the risk of *M.tb* infection, the progression to and the severity of TB disease, mortality, lung cavitation, relapse, accelerates sputum conversion and enhances the efficacy of anti-TB drugs (181–186). Several possible mechanisms for the advantageous effects of metformin have been suggested, including enhancing phagocytosis, phagolysosome fusion and autophagy to increase *M.tb* killing in macrophages; the upregulation of mitochondrial ROS production and intracellular antimycobacterial responses; an anti-inflammatory effect to reduce deleterious inflammation (182, 187, 188). Recent work suggests that metformin also educates CD8+ T-cells which results in increased mitochondrial mass, oxidative phosphorylation, and fatty acid oxidation. Such reprogramming of immune-metabolic circuits increases survival capacity and anti-mycobacterial properties of CD8+ T-cells, as seen by enhancement of BCG vaccine protective efficacy and improvements in the sterilizing ability of antibiotics (189). Since butyrate increases susceptibility to TB (134), increasing its concentration by altering gut microbiota might offer another HDT against *M.tb*. Eicosanoids have also been proposed as potential drug candidates for treating TB. In elegant mice experiments, IL-1 induced PGE2 production which promoted *M.tb* control by suppressing type I IFN (85). Furthermore, PGE2 administration resulted in decreased pulmonary *M.tb* load and associated pathology, as well as increased animal survival (85). These promising findings need to be validated in humans and recent evidence highlights the potential of PGE2 as an HDT candidate for treating TB (190). However, a comprehensive understanding of PGE2 kinetics and its effects on the overall immune system will be needed before eicosanoids can be clinically applied (191).

Similar to the action of metformin in targeting whole-body metabolism, statin intake has been associated with significantly reduced risk of developing TB in both T2D patients and non-diabetic general populations (192). Statins reduce inflammation, modulate the immune responses and have direct antimicrobial effects (193). Used as an adjunct, statins enhance the bactericidal activity of first-line TB drugs against intracellular *M.tb* and shortens TB treatment duration (194). Mechanistic studies indicate that statin-mediated reduction in cholesterol levels within phagosomal membranes counteract *M.tb*-induced inhibition of phagosomal maturation and promotes host-induced autophagy, therefore augmenting host responses against *M.tb* (195).

The role of vitamins in host immunity to *M.tb* are of increasing interest. The active form of vitamin A, *all*-*trans* retinoic acid (ATRA), has been shown to promote autophagy. This results in a reduced bacterial burden in human macrophages infected with *M.tb*, which is induced by cytosolic sensing of double-stranded DNA *via* the STING/TBK1/IRF3 axis (196). Furthermore, ATRA induces a reduction in total cellular cholesterol concentration and promotes lysosomal acidification in *M.tb*-infected monocytes *via* an NPC2-dependent mechanism which results in enhanced antimicrobial activity (197). The possible benefit derived from sunlight and vitamin D supplementation was first suggested in the pre-antibiotic era and has shown to modulate both innate and adaptive immune responses. The biologically active form of vitamin D, 1,25-dihydroxy- vitamin D3 (1,25(OH)2D3), enhances the expression of LL-37 in macrophages, the only cathelicidin-derived antimicrobial peptide found in humans, which promotes the destruction of *M.tb* and consequently autophagy (198, 199). While a series of clinical trials have failed to show vitamin D supplementation impacts on clinical outcomes in TB, there is some evidence that high-dose vitamin D improves the resolution of inflammatory responses during TB therapy and may be beneficial in a subset of TB patients who have a specific polymorphism in the vitamin D receptor (VDR) (200, 201).

The exploration of possible roles of immunomodulatory macronutrient has, to date, focused on glutamine, arginine and tryptophan. Glutamine pathway genes are differentially expressed in *M.tb*-infected macrophages and in the blood of individuals with LTBI or active TB. Glutamine has been identified as the main nitrogen *M.tb* source within infected macrophages (202). Inhibiting glutaminolysis or reducing the availability of glutamine impairs the production of key cytokines by T-cells (IFNγ, IL-1β, IL-17 and IL-22) in response to challenge with *M.tb*, as does genetic polymorphisms in glutamine metabolism genes (including GLS2, SLC1A5, and SLC7A5) (203). Similarly, L-citrulline and L-arginine are necessary for antimycobacterial responses, mediated by microbicidal NO production *via* inducible NO synthase–mediated L-arginine metabolism in infected macrophages. It has been suggested that targeting this pathway might provide novel approaches for enhancing immunity in mycobacterial disease (204). Interestingly, Arg1 conditional gene-deleted mice presented decreased *M.tb* burden compared to wild types, probably due to enhanced macrophage *M.tb* killing capacities (205). In contrast, L-Arg has been shown to contribute to macrophage *M.tb* clearance (206). Observed differences could be explained by different location of Arg1+ macrophages within the granuloma, as well as their role in different infection stages (207). Finally, tryptophan biosynthesis by mycobacteria under stress conditions has been shown to protect *M.tb* from CD4+ T-cell-mediated killing by IFNγ. Inhibition of *M.tb* tryptophan synthesis by the small-molecule 2-amino-6-fluorobenzoic acid (6-FABA) converts *M.tb* into a tryptophan auxotroph and restores the efficacy of failed CD4+ T-cell-mediated host defence (208). Interestingly, the indole propionic acid (IPA), produced by the gut microbiota, also blocks tryptophan biosynthesis in *M.tb via* inhibition of anthranilate synthase (TrpE) which catalyses the first committed step in the tryptophan biosynthesis pathway, by mimicking the physiological allosteric inhibitor of this enzyme (209). Therefore, in contrast to macronutrient supplementation by glutamine and arginine, tryptophan depletion may have a role in *M.tb* control. In the specific context of macrophages, *M.tb* infection causes indoleamine 2,3-dioxygenase (IDO) upregulation, the first rate-limiting enzyme of tryptophan catabolism (210, 211). This not only decreases tryptophan concentrations, but also produces metabolites which activate the aryl hydrocarbon receptor (AHR), which in turns modulate immune activity. For instance, Ahr^-/-^ mice were unable to control *M.tb* H37Rv infection (212). Excellent recent reviews have further covered the potential intervention of metabolic pathways for HDT against TB (213–216).

Treating infected macrophages with statins reduces the viability of intracellular *M. leprae*, similar to that seen with *M.tb*, raising the possible use of statins as an adjuvant HDT for leprosy (217). Also similar to *M.tb*, autophagy is an exciting possible target for HDT leprosy as it promotes bacterial clearance and antigen presentation (218, 219). Not only does autophagy clear *M. leprae* from macrophages but recent evidence suggests it can modulate leprosy disease presentation, driving paucibacillary tuberculoid leprosy in individuals with more autophagic control with a predominance of IL-26, IFNγ, and TNF, autophagy-inducing cytokines (51, 220–223). Rapamycin, metformin and the antiprotozoal drug nitazoxanide have been proposed as HDTs against leprosy *via* modulating autophagic mechanisms to promote the antimicrobial response against *M.leprae* and decrease inflammation-mediated immunopathology (93).

## Conclusion

Mycobacterial infections still represent a major public health issue and we need better treatments. A greater understanding of the host-induced metabolic and immune responses to mycobacterial challenge will aid the design of novel, and needed, host-directed therapeutic strategies. Macrophages are mycobacterial targets and constitute a heterogenous cell population; as reflected by their metabolic diversity and plasticity. Mycobacterial infection shapes host cell metabolism and there are clear metabolic and coupled functional phenotypes associated with particular infection outcomes (ie. M1-like favours infection control, while M2-like macrophages are more permissive to infection). Changes in macrophage polarisation upon infection directly impact the concentration of certain intracellular metabolites. They have the potential to post-translationally modify proteins, including histones. Therefore, changes in host cell metabolism can change the epigenetic landscape of the cell, with long term consequences regarding the host ability to control mycobacterial infection (107, 108). Mycobacterial diseases including TB and leprosy do not exist in isolation. Co-morbidities such as smoking, diabetes and HIV infection can worsen TB outcomes, and this can partially be explained by disease-induced metabolic changes. Gaining mechanistic understanding of this phenomenon brings us closer to the design of new and effective therapies, including expanding the use of current drugs such as metformin. There has been outstanding research in the last decades unravelling the links between cell metabolism and induced immune responses. This is a promising avenue that needs pursuing as it holds great potential to identify new targets for therapy that will aid the fight against ancient and devastating epidemics such as TB and leprosy.

## Author Contributions

AL and CM conceptualised the manuscript. AL, MD, JB, CD, MO’S, and CM wrote the manuscript. All authors contributed to the article and approved the submitted version.

## Funding

AL is supported by the European Commission (H2020-761 MSCA-IF-2018, 841729). CM is supported by the Medical Research Council (MR/T016736/1) and by a Professorial Fellowship and Translational Funds from the University of Birmingham.

## Conflict of Interest

The authors declare that the research was conducted in the absence of any commercial or financial relationships that could be construed as a potential conflict of interest.

## Publisher’s Note

All claims expressed in this article are solely those of the authors and do not necessarily represent those of their affiliated organizations, or those of the publisher, the editors and the reviewers. Any product that may be evaluated in this article, or claim that may be made by its manufacturer, is not guaranteed or endorsed by the publisher.

## References

[1] Zink AR Sola C Reischl U Grabner W Rastogi N Wolf H. Characterization of Mycobacterium Tuberculosis Complex Dnas From Egyptian Mummies by Spoligotyping. J Clin Microbiol (2003) 41(1):359–67. doi: 10.1128/JCM.41.1.359-367.2003 PMC149558 12517873

[2] World Health Organization. Global Tuberculosis Report, Vol. 2020. (2020).

[3] Thekkur P Tweya H Phiri S Mpunga J Kalua T Kumar AMV. Assessing the Impact of Covid-19 on Tb and Hiv Programme Services in Selected Health Facilities in Lilongwe, Malawi: Operational Research in Real Time. Trop Med Infect Dis (2021) 6(2):1–16. doi: 10.3390/tropicalmed6020081 PMC8163191 34069332

[4] World Health Organization. Towards Zero Leprosy Towards Zero Leprosy - Global Leprosy (Hansen’s Disease) Strategy 2021–2030 (2021). Available at: https://www.who.int/publications/i/item/9789290228509.

[5] Deps P Geurra P Nasser S Simon M. Hemolytic Anemia in Patients Receiving Daily Dapsone for the Treatment of Leprosy. Lepr Rev (2012) 83(3):305–7. doi: 10.47276/lr.83.3.305 23356031

[6] Achkar JM Jenny-Avital ER. Incipient and Subclinical Tuberculosis: Defining Early Disease States in the Context of Host Immune Response. J Infect Dis (2011) 204(SUPPL. 4):1179–86. doi: 10.1093/infdis/jir451 PMC3192549 21996700

[7] Pai M Behr MA Dowdy D Dheda K Divangahi M Boehme CC. Tuberculosis. Nat Rev Dis Prim (2016) 2:1–23. doi: 10.1038/nrdp.2016.76 27784885

[8] Gaschignard J Grant AV Van Thuc N Orlova M Cobat A Huong NT. Pauci- and Multibacillary Leprosy: Two Distinct, Genetically Neglected Diseases. PloS Negl Trop Dis (2016) 10(5):1–20. doi: 10.1371/journal.pntd.0004345 PMC4878860 27219008

[9] da Silva Prata RB de mattos Barbosa MG de Andrade Silva BJ Paixao de Oliveira JA Lameira Bittencourt T Olmo Pinheiro R. Macrophages in the Pathogenesis of Leprosi. IntechOpen (2016) 1:1–19. doi: 10.5772/intechopen.88754

[10] Tortoli E Fedrizzi T Meehan CJ Trovato A Grottola A Giacobazzi E. The New Phylogeny of the Genus Mycobacterium: The Old and the News. Infect Genet Evol (2017) 56(October):19–25. doi: 10.1016/j.meegid.2017.10.013 29030295

[11] Demangel C. Immunity Against Mycobacterium Ulcerans: The Subversive Role of Mycolactone. Immunol Rev (2021) 301(1):209–21. doi: 10.1111/imr.12956 33607704

[12] Omansen TF Erbowor-Becksen A Yotsu R van der Werf TS Tiendrebeogo A Grout L. Global Epidemiology of Buruli Ulcer, 2010–2017, and Analysis of 2014 WHO Programmatic Targets. Emerg Infect Dis (2019) 25(12)::2183–90. doi: 10.3201/eid2512.190427 PMC6874257 31742506

[13] Kasperbauer SH Daley CL. Mycobacterium Chimaera Infections Related to the Heater-Cooler Unit Outbreak: A Guide to Diagnosis and Management. Clin Infect Dis (2019) 68(7):1244–50. doi: 10.1093/cid/ciy789 30371755

[14] Leto Barone A Grzelak M Frost C Ngagge L Ge S Kolegraff K. Atypical Mycobacterial Infections After Plastic Surgery Procedures Abroad: A Multidisciplinary Algorithm for Diagnosis and Treatment. Ann Plast Surg (2020) 84(3):257–62. doi: 10.1097/SAP.0000000000002061 31688120

[15] Martiniano SL Esther CR Haworth CS Kasperbauer SH Zemanick ET Caverly LJ. Challenging Scenarios in Nontuberculous Mycobacterial Infection in Cystic Fibrosis. Pediatr Pulmonol (2020) 55(2):521–5. doi: 10.1002/ppul.24604 PMC6980303 31821718

[16] Tauber AI. Metchnikoff and the Phagocytosis Theory. Nat Rev Mol Cell Biol (2003) 4(11):897–901. doi: 10.1038/nrm1244 14625539

[17] Watanabe S Alexander M Misharin AV Budinger GRS. The Role of Macrophages in the Resolution of Inflammation. J Clin Invest (2019) 129(7):2619–28. doi: 10.1172/JCI124615 PMC6597225 31107246

[18] Murray PJ Wynn TA. Protective and Pathogenic Functions of Macrophage Subsets. Nat Rev Immunol (2011) 11(11):723–37. doi: 10.1038/nri3073 PMC3422549 21997792

[19] Lawrence T Natoli G. Transcriptional Regulation of Macrophage Polarization: Enabling Diversity With Identity. Nat Rev Immunol (2011) 11(11):750–61. doi: 10.1038/nri3088 22025054

[20] Xue J Schmidt SV Sander J Draffehn A Krebs W Quester I. Transcriptome-Based Network Analysis Reveals a Spectrum Model of Human Macrophage Activation. Immun (2014) 40(2):274–88. doi: 10.1016/j.immuni.2014.01.006 PMC3991396 24530056

[21] Martinez FO Gordon S. The M1 and M2 Paradigm of Macrophage Activation: Time for Reassessment. F1000Prime Rep (2014) 6:1–13. doi: 10.12703/P6-13 24669294 PMC3944738

[22] O’Neill LAJ Pearce EJ. Immunometabolism Governs Dendritic Cell and Macrophage Function. J Exp Med (2016) 213(1):15–23. doi: 10.1084/jem.20151570 26694970 PMC4710204

[23] Cohen SB Gern BH Delahaye JL Adams KN Plumlee CR Winkler JK. Alveolar Macrophages Provide an Early Mycobacterium Tuberculosis Niche and Initiate Dissemination. Cell Host Microbe (2018) 24(3):439–46 e4. doi: 10.1016/j.chom.2018.08.001 30146391 PMC6152889

[24] Davey T Rees R. The Nasal Dicharge in Leprosy: Clinical and Bacteriological Aspects. Lepr Rev (1974) 45(2):121–34. doi: 10.5935/0305-7518.19740014 4608620

[25] Scollard DM Adams LB Gillis TP Krahenbuhl JL Truman RW Williams DL. The Continuing Challenges of Leprosy. Clin Microbiol Rev (2006) 19(2):338–81. doi: 10.1128/CMR.19.2.338-381.2006 PMC1471987 16614253

[26] Bratschi MW Steinmann P Wickenden A Gillis TP. Current Knowledge on Mycobacterium Leprae Transmission: A Systematic Literature Review. Lepr Rev (2015) 86(2):142–55. doi: 10.47276/lr.86.2.142 26502685

[27] Torrado E Fraga AG Castro AG Stragier P Meyers WM Portaels F. Evidence for an Intramacrophage Growth Phase of Mycobacterium Ulcerans. Infect Immun (2007) 75(2):977–87. doi: 10.1128/IAI.00889-06 PMC1828495 17145944

[28] Philips JA Ernst JD. Tuberculosis Pathogenesis and Immunity. Annu Rev Pathol Mech Dis (2012) 7:353–84. doi: 10.1146/annurev-pathol-011811-132458 22054143

[29] Marakalala MJ Martinez FO Plüddemann A Gordon S. Macrophage Heterogeneity in the Immunopathogenesis of Tuberculosis. Front Microbiol (2018) 9:1–15. doi: 10.3389/fmicb.2018.01028 29875747 PMC5974223

[30] Huang L Nazarova EV Tan S Liu Y Russell DG. Growth of Mycobacterium Tuberculosis In Vivo Segregates With Host Macrophage Metabolism and Ontogeny. J Exp Med (2018) 215(4):1135–52. doi: 10.1084/jem.20172020 PMC5881470 29500179

[31] Gordon S. Alternative Activation of Macrophages. Nat Rev Immunol (2003) 3(1):23–35. doi: 10.1038/nri978 12511873

[32] Guirado E Schlesinger LS Kaplan G. Macrophages in Tuberculosis: Friend or Foe. Semin Immunopathol (2013) 35:563–83. doi: 10.1007/s00281-013-0388-2 PMC3763202 23864058

[33] Gleeson LE O’Leary SM Ryan D McLaughlin AM Sheedy FJ Keane J. Cigarette Smoking Impairs the Bioenergetic Immune Response to Mycobacterium Tuberculosis Infection. Am J Respir Cell Mol Biol (2018) 59(5):572–9. doi: 10.1165/rcmb.2018-0162OC 29944387

[34] Tan SYS Krasnow MA. Developmental Origin of Lung Macrophage Diversity. Dev (2016) 143(8):1318–27. doi: 10.1242/dev.129122 PMC4852511 26952982

[35] Gibbings SL Thomas SM Atif SM McCubbrey AL Desch AN Danhorn T. Three Unique Interstitial Macrophages in the Murine Lung at Steady State. Am J Respir Cell Mol Biol (2017) 57(1):66–76. doi: 10.1165/rcmb.2016-0361OC 28257233 PMC5516280

[36] Srivastava S Ernst JD Desvignes L. Beyond Macrophages: The Diversity of Mononuclear Cells in Tuberculosis. Immunol Rev (2014) 262(1):179–92. doi: 10.1111/imr.12217 PMC4203409 25319335

[37] Russell DG Cardona PJ Kim MJ Allain S Altare F. Foamy Macrophages and the Progression of the Human Tuberculosis Granuloma. Nat Immunol (2009) 10(9):943–8. doi: 10.1038/ni.1781 PMC2759071 19692995

[38] Chambers T Spector W. Inflammatory Giant Cells. Immunobiology (1982) 161((3-4):283–9. doi: 10.1016/S0171-2985(82)80084-3 7047375

[39] Moura DF de Mattos KA Amadeu TP Andrade PR Sales JS Schmitz V. CD163 Favors Mycobacterium Leprae Survival and Persistence by Promoting Anti-Inflammatory Pathways in Lepromatous Macrophages. Eur J Immunol (2012) 42(11):2925–36. doi: 10.1002/eji.201142198 22851198

[40] Guarner J. Buruli Ulcer : Review of a Neglected Skin Mycobacterial. J Clin Microbiol (2018) 2):1–8. doi: 10.1128/JCM.01507-17 PMC5869816 29343539

[41] Baron L Paatero AO Morel JD Impens F Guenin-Macé L Saint-Auret S. Mycolactone Subverts Immunity by Selectively Blocking the Sec61 Translocon. J Exp Med (2016) 213(13):2885–96. doi: 10.1084/jem.20160662 PMC5154940 27821549

[42] Demangel C High S. Sec61 Blockade by Mycolactone: A Central Mechanism in Buruli Ulcer Disease. Biol Cell (2018) 110(11):237–48. doi: 10.1111/boc.201800030 30055020

[43] McKenna M Simmonds RE High S. Mechanistic Insights Into the Inhibition of Sec61-Dependent Co- and Post-Translational Translocation by Mycolactone. J Cell Sci (2016) 129(7):1404–15. doi: 10.1242/jcs.182352 PMC4852723 26869228

[44] McKenna M Simmonds RE High S. Mycolactone Reveals the Substrate-Driven Complexity of Sec61-Dependent Transmembrane Protein Biogenesis. J Cell Sci (2017) 130(7):1307–20. doi: 10.1242/jcs.198655 PMC5399781 28219954

[45] Hall BS Hill K McKenna M Ogbechi J High S Willis AE. The Pathogenic Mechanism of the Mycobacterium Ulcerans Virulence Factor, Mycolactone, Depends on Blockade of Protein Translocation Into the ER. PloS Pathog (2014) 10(4):15–7. doi: 10.1371/journal.ppat.1004061 PMC3974873 24699819

[46] Morel JD Paatero AO Wei J Yewdell JW Guenin-Macé L Van Haver D. Proteomics Reveals Scope of Mycolactone-Mediated Sec61 Blockade and Distinctive Stress Signature. Mol Cell Proteomics (2018) 17(9):1750–65. doi: 10.1074/mcp.RA118.000824 PMC6126388 29915147

[47] Gleeson LE Sheedy FJ Palsson-McDermott EM Triglia D O’Leary SM O’Sullivan MP. Cutting Edge: Mycobacterium Tuberculosis Induces Aerobic Glycolysis in Human Alveolar Macrophages That Is Required for Control of Intracellular Bacillary Replication. J Immunol (2016) 196(6):2444–9. doi: 10.4049/jimmunol.1501612 26873991

[48] Cumming BM Addicott KW Adamson JH Steyn AJ. Mycobacterium Tuberculosis Induces Decelerated Bioenergetic Metabolism in Human Macrophages. Elife (2018) 7:1–28. doi: 10.7554/eLife.39169 PMC6286123 30444490

[49] Hackett EE Charles-Messance H O’Leary SM Gleeson LE Munoz-Wolf N Case S. Mycobacterium Tuberculosis Limits Host Glycolysis and IL-1beta by Restriction of PFK-M via MicroRNA-21. Cell Rep (2020) 30(1):124–136 e4. doi: 10.1016/j.celrep.2019.12.015 31914380 PMC7764301

[50] Olson GS Murray TA Jahn AN Mai D Diercks AH Gold ES. Type I Interferon Decreases Macrophage Energy Metabolism During Mycobacterial Infection. Cell Rep (2021) 35(9):109195. doi: 10.1016/j.celrep.2021.109195 34077724 PMC8244443

[51] Yamamura M. Erratum: Defining Protective Responses to Pathogens: Cytokine Profiles in Leprosy Lesions (Science (277)). Science (1992) 255(5040):12. doi: 10.1126/science.1925582 1553522

[52] Fulco T de O Andrade PR Barbosa MG de M Pinto TGT Ferreira PF Ferreira H. Effect of Apoptotic Cell Recognition on Macrophage Polarization and Mycobacterial Persistence. Infect Immun (2014) 82(9):3968–78. doi: 10.1128/IAI.02194-14 PMC4187838 25024361

[53] Chandra P He L Zimmerman M Yang G Köster S Ouimet M. Inhibition of Fatty Acid Oxidation Promotes Macrophage Control of Mycobacterium Tuberculosis. MBio (2020) 11(4):1–15. doi: 10.1128/mBio.01139-20 PMC7343992 32636249

[54] Montoya D Cruz D Teles RMB Lee DJ Ochoa MT Krutzik SR. Divergence of Macrophage Phagocytic and Antimicrobial Programs in Leprosy. Cell Host Microbe (2009) 6(4):343–53. doi: 10.1016/j.chom.2009.09.002 PMC2764558 19837374

[55] Yang D Shui T Miranda JW Gilson DJ Song Z Chen J. Mycobacterium Leprae-Infected Macrophages Preferentially Primed Regulatory T Cell Responses and Was Associated With Lepromatous Leprosy. PloS Negl Trop Dis (2016) 10(1):1–13. doi: 10.1371/journal.pntd.0004335 PMC4713426 26751388

[56] Tannahill GM Curtis AM Adamik J Palsson-Mcdermott EM McGettrick AF Goel G. Succinate is an Inflammatory Signal That Induces IL-1β Through HIF-1α. Nature (2013) 496(7444):238–42. doi: 10.1038/nature11986 PMC4031686 23535595

[57] Braverman J Sogi KM Benjamin D Nomura DK Stanley SA. Hif-1α is an Essential Mediator of IFN-γ–Dependent Immunity to Mycobacterium Tuberculosis. J Immunol (2016) 197(4):1287–97. doi: 10.4049/jimmunol.1600266 PMC4976004 27430718

[58] Lachmandas E Beigier-Bompadre M Cheng SC Kumar V van Laarhoven A Wang X. Rewiring Cellular Metabolism *via* the AKT/mTOR Pathway Contributes to Host Defence Against Mycobacterium Tuberculosis in Human and Murine Cells. Eur J Immunol (2016) 46(11):2574–86. doi: 10.1002/eji.201546259 PMC5129526 27624090

[59] Cumming BM Pacl HT Steyn AJC. Relevance of the Warburg Effect in Tuberculosis for Host-Directed Therapy. Front Cell Infect Microbiol (2020) 10(506):576596. doi: 10.3389/fcimb.2020.576596 33072629 PMC7531540

[60] Hackett EE Sheedy FJ. An Army Marches on its Stomach: Metabolic Intermediates as Antimicrobial Mediators in Mycobacterium Tuberculosis Infection. Front Cell Infect Microbiol (2020) 10(446):446. doi: 10.3389/fcimb.2020.00446 32984072 PMC7477320

[61] Sheedy FJ Divangahi M. Targeting Immunometabolism in Host Defence Against Mycobacterium Tuberculosis. Immunology (2021) 162(2):145–59. doi: 10.1111/imm.13276 PMC7808148 33020911

[62] Pisu D Huang L Narang V Theriault M Lê-Bury G Lee B. Single Cell Analysis of M. Tuberculosis Phenotype and Macrophage Lineages in the Infected Lung. J Exp Med (2021) 218(9):1–25. doi: 10.1084/jem.20210615 PMC8302446 34292313

[63] Mills EL Kelly B Logan A Costa ASH Varma M Bryant CE. Succinate Dehydrogenase Supports Metabolic Repurposing of Mitochondria to Drive Inflammatory Macrophages. Cell (2016) 167(2):457–470.e13. doi: 10.1016/j.cell.2016.08.064 27667687 PMC5863951

[64] Shi L Jiang Q Bushkin Y Subbian S Tyagi S. Biphasic Dynamics of Macrophage Immunometabolism During Mycobacterium Tuberculosis Infection. MBio (2019) 10(2):1–19. doi: 10.1128/mBio.02550-18 PMC6437057 30914513

[65] Mills EL Ryan DG Prag HA Dikovskaya D Menon D Zaslona Z. Itaconate Is an Anti-Inflammatory Metabolite That Activates Nrf2 via Alkylation of KEAP1. Nature (2018) 556(7699):113–7. doi: 10.1038/nature25986 PMC6047741 29590092

[66] Lampropoulou V Sergushichev A Bambouskova M Nair S Vincent EE Loginicheva E. Itaconate Links Inhibition of Succinate Dehydrogenase With Macrophage Metabolic Remodeling and Regulation of Inflammation. Cell Metab (2016) 24(1):158–66. doi: 10.1016/j.cmet.2016.06.004 PMC5108454 27374498

[67] Michelucci A Cordes T Ghelfi J Pailot A Reiling N Goldmann O. Immune-Responsive Gene 1 Protein Links Metabolism to Immunity by Catalyzing Itaconic Acid Production. Proc Natl Acad Sci USA (2013) 110(19):7820–5. doi: 10.1073/pnas.1218599110 PMC3651434 23610393

[68] Hoffmann E Machelart A Belhaouane I Deboosere N Pauwels A-M Saint-André J-P. IRG1 Controls Immunometabolic Host Response and Restricts Intracellular Mycobacterium Tuberculosis Infection. bioRxiv (2019), 1–40. doi: 10.1101/761551

[69] Nair S Huynh JP Lampropoulou V Loginicheva E Esaulova E Gounder AP. Irg1 Expression in Myeloid Cells Prevents Immunopathology During *M. tuberculosis* Infection. J Exp Med (2018) 215(4):1035–45. doi: 10.1084/jem.20180118 PMC5881474 29511063

[70] Batista-Silva LR Rodrigues LS Vivarini ADC Costa FDMR De Mattos KA Costa MRSN. Mycobacterium Leprae-Induced Insulin-Like Growth Factor I Attenuates Antimicrobial Mechanisms, Promoting Bacterial Survival in Macrophages. Sci Rep (2016) 6(May):1–13. doi: 10.1038/srep27632 27282338 PMC4901318

[71] Medeiros RCA De Vasconcelos Girardi KDC Cardoso FKL De Siqueira Mietto B De Toledo Pinto TG Gomez LS. Subversion of Schwann Cell Glucose Metabolism by Mycobacterium Leprae. J Biol Chem (2016) 291(41):21375–87. doi: 10.1074/jbc.M116.725283 PMC5076808 27555322

[72] Borah K Girardi DV Mendum TA Santos M Beste DJV Lara A. Intracellular Mycobacterium Leprae Utilizes Host Glucose as a Carbon Source in Schwann Cells. Am Soc Microbiol (2019) 10(6):1–9. doi: 10.1128/mBio.02351-19 PMC6918074 31848273

[73] Fallows D Peixoto B Kaplan G Manca C. Mycobacterium Leprae Alters Classical Activation of Human Monocytes In Vitro. J Inflamm (United Kingdom) (2016) 13(1):4–8. doi: 10.1186/s12950-016-0117-4 PMC4788835 26973434

[74] Ma Y Pei Q Zhang L Lu J Shui T Chen J. Live Mycobacterium Leprae Inhibits Autophagy and Apoptosis of Infected Macrophages and Prevents Engulfment of Host Cell by Phagocytes. Am J Transl Res (2018) 10(9):2929–39. PMC6176229 30323879

[75] Sarfo FS Chevalier F Aka N Phillips RO Amoako Y Boneca IG. Mycolactone Diffuses Into the Peripheral Blood of Buruli Ulcer Patients - Implications for Diagnosis and Disease Monitoring. PloS Negl Trop Dis (2011) 5(7):1–8. doi: 10.1371/journal.pntd.0001237 PMC3139662 21811642

[76] Hong H Coutanceau E Leclerc M Caleechurn L Leadlay PF Demangel C. Mycolactone Diffuses From Mycobacterium Ulcerans-Infected Tissues and Targets Mononuclear Cells in Peripheral Blood and Lymphoid Organs. PloS Negl Trop Dis (2008) 2(10):1–8. doi: 10.1371/journal.pntd.0000325 PMC2565835 18941518

[77] Niang F Sarfo FS Frimpong M Guenin-Macé L Wansbrough-Jones M Stinear T. Metabolomic Profiles Delineate Mycolactone Signature in Buruli Ulcer Disease. Sci Rep (2015) 5:1–14. doi: 10.1038/srep17693 PMC4669498 26634444

[78] Laval T Chaumont L Demangel C. Not Too Fat to Fight: The Emerging Role of Macrophage Fatty Acid Metabolism in Immunity to Mycobacterium Tuberculosis. Immunol Rev (2021) 301(1):84–97. 10.1111/imr.12952 33559209

[79] Peyron P Vaubourgeix J Poquet Y Levillain F Botanch C Bardou F. Foamy Macrophages From Tuberculous Patients’ Granulomas Constitute a Nutrient-Rich Reservoir for M. Tuberculosis Persistence. PloS Pathog (2008) 4(11):1–14. doi: 10.1371/journal.ppat.1000204 PMC2575403 19002241

[80] Elamin AA Stehr M Singh M. Lipid Droplets and Mycobacterium Leprae Infection. J Pathog (2012) 2012:1–10. doi: 10.1155/2012/361374 PMC3503283 23209912

[81] Daniel J Maamar H Deb C Sirakova TD Kolattukudy PE. Mycobacterium Tuberculosis Uses Host Triacylglycerol to Accumulate Lipid Droplets and Acquires a Dormancy-Like Phenotype in Lipid-Loaded Macrophages. PloS Pathog (2011) 7(6):1–16. doi: 10.1371/journal.ppat.1002093 PMC3121879 21731490

[82] Knight M Braverman J Asfaha K Gronert K Stanley S. Lipid Droplet Formation in Mycobacterium Tuberculosis Infected Macrophages Requires IFN-γ/Hif-1α Signaling and Supports Host Defense. PloS Pathog (2018) 14(1):e1006874. doi: 10.1371/journal.ppat.1006874 29370315 PMC5800697

[83] Mayer-Barber KD Sher A. Cytokine and Lipid Mediator Networks in Tuberculosis. Immunol Rev (2015) 264(1):264–75. doi: 10.1111/imr.12249 PMC4339232 25703565

[84] Bosch M Sánchez-Álvarez M Fajardo A Kapetanovic R Steiner B Dutra F. Mammalian Lipid Droplets Are Innate Immune Hubs Integrating Cell Metabolism and Host Defense. Science (2020) 370(6514):1–12. doi: 10.1126/science.aay8085 33060333

[85] Mayer-Barber KD Andrade BB Oland SD Amaral EP Barber DL Gonzales J. Host-Directed Therapy of Tuberculosis Based on Interleukin-1 and Type I Interferon Crosstalk. Nat (2014) 511(7507):99–103. doi: 10.1038/nature13489 PMC4809146 24990750

[86] Sorgi CA Soares EM Rosada RS Bitencourt CS Zoccal KF Pereira PAT. Eicosanoid Pathway on Host Resistance and Inflammation During Mycobacterium Tuberculosis Infection Is Comprised by LTB4 Reduction But Not PGE2 Increment. Biochim Biophys Acta - Mol Basis Dis (2020) 1866(3):165574. doi: 10.1016/j.bbadis.2019.165574 31666208

[87] Nore KG Jørgensen MJ Dyrhol-Riise AM Jenum S Tonby K. Elevated Levels of Anti-Inflammatory Eicosanoids and Monocyte Heterogeneity in Mycobacterium Tuberculosis Infection and Disease. Front Immunol (2020) 11(November):1–12. doi: 10.3389/fimmu.2020.579849 33304347 PMC7693556

[88] Oliveira MF Medeiros RCA Mietto BS Calvo TL Mendonça APM Rosa TLSA. Reduction of Host Cell Mitochondrial Activity as Mycobacterium Leprae’s Strategy to Evade Host Innate Immunity. Immunol Rev (2021) 301(1):193–208. doi: 10.1111/imr.12962 33913182 PMC10084840

[89] Mattos KA Oliveira VCG Berrêdo-Pinho M Amaral JJ Antunes LCM Melo RCN. Mycobacterium Leprae Intracellular Survival Relies on Cholesterol Accumulation in Infected Macrophages: A Potential Target for New Drugs for Leprosy Treatment. Cell Microbiol (2014) 16(6):797–815. doi: 10.1111/cmi.12279 24552180 PMC4262048

[90] Cruz D Watson AD Miller CS Montoya D Ochoa MT Sieling PA. Host-Derived Oxidized Phospholipids and HDL Regulate Innate Immunity in Human Leprosy. J Clin Invest (2008) 118(8):2917–28. doi: 10.1172/JCI34189 PMC2467381 18636118

[91] Kaur G Kaur J. Multifaceted Role of Lipids in Mycobacterium Leprae. Future Microbiol (2017) 12(4):315–35. doi: 10.2217/fmb-2016-0173 28287297

[92] Marques MAM Berrêdo-Pinho M Rosa TLSA Pujari V Lemes RMR Lery LMS. The Essential Role of Cholesterol Metabolism in the Intracellular Survival of Mycobacterium Leprae Is Not Coupled to Central Carbon Metabolism and Energy Production. J Bacteriol (2015) 197(23):3698–707. doi: 10.1128/JB.00625-15 PMC4626898 26391209

[93] Toledo Pinto TG Batista-Silva LR Medeiros R Lara FA Moraes M. Type I Interferons, Autophagy and Host Metabolism in Leprosy. Front Immunol (2018) 9(APR):1–11. doi: 10.3389/fimmu.2018.00806 29755459 PMC5932357

[94] Wilburn KM Fieweger RA VanderVen BC. Cholesterol and Fatty Acids Grease the Wheels of Mycobacterium Tuberculosis Pathogenesis. Pathog Dis (2018) 76(2):1–14. doi: 10.1093/femspd/fty021 PMC6251666 29718271

[95] Neyrolles O Wolschendorf F Mitra A Niederweis M. Mycobacteria, Metals, and the Macrophage. Immunol Rev (2015) 264(1):249–63. doi: 10.1111/imr.12265 PMC4521620 25703564

[96] Pisu D Huang L Grenier JK Russell DG. Dual RNA-Seq of Mtb-Infected Macrophages *In Vivo* Reveals Ontologically Distinct Host-Pathogen Interactions. Cell Rep (2020) 30(2):335–350.e4. doi: 10.1016/j.celrep.2019.12.033 31940480 PMC7032562

[97] de Mattos Barbosa MG da Silva Prata RB Andrade PR Ferreira H de Andrade Silva BJ da Paixão de Oliveira JA. Indoleamine 2,3-Dioxygenase and Iron Are Required for Mycobacterium Leprae Survival. Microbes Infect (2017) 19(11):505–14. doi: 10.1016/j.micinf.2017.06.006 28684130

[98] Prabakaran S Lippens G Steen H Gunawardena J. Post-Translational Modification: Nature’s Escape From Genetic Imprisonment and the Basis for Dynamic Information Encoding. Wiley Interdiscip Rev Syst Biol Med (2012) 4(6):565–83. doi: 10.1002/wsbm.1185 PMC3473174 22899623

[99] Karve TM Cheema AK. Small Changes Huge Impact: The Role of Protein Posttranslational Modifications in Cellular Homeostasis and Disease. J Amino Acids (2011) 2011:1–13. doi: 10.4061/2011/207691 PMC3268018 22312457

[100] Liu J Qian C Cao X. Post-Translational Modification Control of Innate Immunity. Immun (2016) 45(1):15–30. doi: 10.1016/j.immuni.2016.06.020 27438764

[101] Allis CD Jenuwein T. The Molecular Hallmarks of Epigenetic Control. Nat Rev Genet (2016) 17(8):487–500. doi: 10.1038/nrg.2016.59 27346641

[102] Zhang D Tang Z Huang H Zhou G Cui C Weng Y. Metabolic Regulation of Gene Expression by Histone Lactylation. Nature (2019) 574(7779):575–80. doi: 10.1038/s41586-019-1678-1 PMC6818755 31645732

[103] Dichtl S Lindenthal L Zeitler L Behnke K Schlösser D Strobl B. Lactate and IL6 Define Separable Paths of Inflammatory Metabolic Adaptation. Scie Adv (2021) 7(26):1–11. doi: 10.1126/sciadv.abg3505 PMC8221612 34162546

[104] Llibre A Grudzinska FS O’Shea MK Duffy D Thickett DR Mauro C. Lactate Crosstalk in Host-Pathogen Interactions. Biochem J (2021) 478(17):3157–78. doi: 10.1042/BCJ20210263 PMC8454702 34492096

[105] Netea MG Domínguez-Andrés J Barreiro LB Chavakis T Divangahi M Fuchs E. Defining Trained Immunity and its Role in Health and Disease. Nat Rev Immunol (2020) 20(6):375–88. doi: 10.1038/s41577-020-0285-6 PMC7186935 32132681

[106] Chao WC Yen CL Wu YH Chen SY Hsieh CY Chang TC. Increased Resistin may Suppress Reactive Oxygen Species Production and Inflammasome Activation in Type 2 Diabetic Patients With Pulmonary Tuberculosis Infection. Microbes Infect (2015) 17(3):195–204. doi: 10.1016/j.micinf.2014.11.009 25528597

[107] Arts RJW Carvalho A La Rocca C Palma C Rodrigues F Silvestre R. Immunometabolic Pathways in BCG-Induced Trained Immunity. Cell Rep (2016) 17(10):2562–71. doi: 10.1016/j.celrep.2016.11.011 PMC5177620 27926861

[108] Khan N Downey J Sanz J Kaufmann E Blankenhaus B Pacis A. M. Tuberculosis Reprograms Hematopoietic Stem Cells to Limit Myelopoiesis and Impair Trained Immunity. Cell (2020) 183(3):752–770.e22. doi: 10.1016/j.cell.2020.09.062 33125891 PMC7599081

[109] Batzer MA Deininger PL. Alu Repeats and Human Genomic Diversity. Nat Rev Genet (2002) 3(5):370–9. doi: 10.1038/nrg798 11988762

[110] Bouttier M Laperriere D Memari B Mangiapane J Fiore A Mitchell E. Alu Repeats as Transcriptional Regulatory Platforms in Macrophage Responses to M.Tuberculosis Infection. Nucleic Acids Res (2016) 44(22):10571–87. doi: 10.1093/nar/gkw782 PMC5159539 27604870

[111] Maruthai K Subramanian M. Methylation Status of Alu Repetitive Elekents in Children With Tuberculosis Disease. Int J Mycobacteriol (2017) 6(3):239–45. doi: 10.4103/ijmy.ijmy_86_18 30198503

[112] Holt PG Keast D. Environmentally Induced Changes in Immunological Function: Acute and Chronic Effects of Inhalation of Tobacco Smoke and Other Atmospheric Contaminants in Man and Experimental Animals. Bacteriol Rev (1977) 41(1):205–16. doi: 10.1128/br.41.1.205-216.1977 PMC413999 405003

[113] Holt PG. Immune and Inflammatory Function in Cigarette Smokers. Thorax (1987) 42(4):241–9. doi: 10.1136/thx.42.4.241 PMC460693 3303428

[114] Sopori M. Effects of Cigarette Smoke on the Immune System. Nat Rev Immunol (2002) 2:372–7. doi: 10.1038/nri803 12033743

[115] Stämpfli MR Anderson GP. How Cigarette Smoke Skews Immune Responses to Promote Infection, Lung Disease and Cancer. Nat Rev Immunol (2009) 9(5):377–84. doi: 10.1038/nri2530 19330016

[116] Qiu F Liang CL Liu H Zeng YQ Hou S Huang S. Impacts of Cigarette Smoking on Immune Responsiveness: Up and Down or Upside Down? Oncotarget (2017) 8(1):268–84. doi: 10.18632/oncotarget.13613 PMC5352117 27902485

[117] O’Leary SM Coleman MM Chew WM Morrow C McLaughlin AM Gleeson LE. Cigarette Smoking Impairs Human Pulmonary Immunity to Mycobacterium Tuberculosis. Am J Respir Crit Care Med (2014) 190(12):1430–6. doi: 10.1164/rccm.201407-1385OC 25390734

[118] Aridgides DS Mellinger DL Armstrong DA Hazlett HF Dessaint JA Hampton TH. Functional and Metabolic Impairment in Cigarette Smoke-Exposed Macrophages is Tied to Oxidative Stress. Sci Rep (2019) 9(1):1–11. doi: 10.1038/s41598-019-46045-7 31270372 PMC6610132

[119] Buscetta M Di Vincenzo S Miele M Badami E Pace E Cipollina C. Cigarette Smoke Inhibits the NLRP3 Inflammasome and Leads to Caspase-1 Activation via the TLR4-TRIF-Caspase-8 Axis in Human Macrophages. FASEB J (2020) 34(1):1819–32. doi: 10.1096/fj.201901239R 31914643

[120] Stevenson CR Critchley JA Forouhi NG Roglic G Williams BG Dye C. Diabetes and the Risk of Tuberculosis: A Neglected Threat to Public Health? Chronic Illn (2007) 3(3):228–45. doi: 10.1177/1742395307081502 18083679

[121] Dooley KE Chaisson RE. Tuberculosis and Diabetes Mellitus: Convergence of Two Epidemics. Lancet Infect Dis (2009) 9(12):737–46. doi: 10.1016/S1473-3099(09)70282-8 PMC2945809 19926034

[122] Lönnroth K Roglic G Harries AD. Improving Tuberculosis Preven- Tion and Care Through Addressing the Global Diabetes Epidemic: From Evidence to Policy and Practice. Lancet Diabetes Endocrinol (2014) 2(9):730–9. doi: 10.1016/S2213-8587(14)70109-3 25194886

[123] Workneh MH Bjune GA Yimer SA. Prevalence and Associated Factors of Tuberculosis and Diabetes Mellitus Comorbidity: A Systematic Review1. PloS One (2017) 12(4):1–25. doi: 10.1371/journal.pone.0175925 PMC5400500 28430796

[124] Saiki O Negoro S Tsuyuguchi I Yamamura Y. Depressed Immunological Defense Mechanisms in Mice With Experimentally Induced Diabetes. Infect Immun (1980) 28(1):127–31. doi: 10.1128/iai.28.1.127-131.1980 PMC550901 6966615

[125] Yamashiro S Kawakami K Uezu K Kinjo T Miyagi K Nakamura K. Lower Expression of Th1-Related Cytokines and Inducible Nitric Oxide Synthase in Mice With Streptozotocin-Induced Diabetes Mellitus Infected With Mycobacterium Tuberculosis. Clin Exp Immunol (2005) 139(1):57–64. doi: 10.1111/j.1365-2249.2005.02677.x 15606614 PMC1809276

[126] Martens GW Arikan MC Lee J Ren F Greiner D Kornfeld H. Tuberculosis Susceptibility of Diabetic Mice. Am J Respir Cell Mol Biol (2007) 37(5):518–24. doi: 10.1165/rcmb.2006-0478OC PMC2048677 17585110

[127] Sugawara I Mizuno S. Higher Susceptibility of Type 1 Diabetic Rats to Mycobacterium Tuberculosis Infection. Tohoku J Exp Med (2008) 216(4):363–70. doi: 10.1620/tjem.216.363 19060451

[128] Sugawara I Yamada H Mizuno S. Pulmonary Tuberculosis in Spontaneously Diabetic Goto Kakizaki Rats. Tohoku J Exp Med (2004) 204(2):135–45. doi: 10.1620/tjem.204.135 15383694

[129] Podell BK Ackart DF Kirk NM Eck SP Bell C Basaraba RJ. Non-Diabetic Hyperglycemia Exacerbates Disease Severity in Mycobacterium Tuberculosis Infected Guinea Pigs. PloS One (2012) 7(10):1–10. doi: 10.1371/journal.pone.0046824 PMC3464230 23056469

[130] Moutschen M Scheen A Lefebvre P. Impaired Immune Responses in Diabetes Mellitus: Analysis of the Factors and Mechanisms Involved. Relevance to the Increased Susceptibility of Diabetic Patients to Specific Infections. Diabete Metab (1992) 18(3):187–201. 1397473

[131] Stew SS Martinez PJ Schlesinger LS Restrepo BI. Differential Expression of Monocyte Surface Markers Among TB Patients With Diabetes Co-Morbidity. Tuberculosis (2013) 93(SUPPL.):578–82. doi: 10.1016/S1472-9792(13)70015-5 PMC4028445 24388654

[132] Gomez DI Twahirwa M Schlesinger LS Restrepo BI. Reduced Mycobacterium Tuberculosis Association With Monocytes From Diabetes Patients That Have Poor Glucose Control. Tuberculosis (2013) 93(2):192–7. doi: 10.1016/j.tube.2012.10.003 PMC3580120 23131496

[133] Vallerskog T Martens GW Kornfeld H. Diabetic Mice Display a Delayed Adaptive Immune Response to Mycobacterium Tuberculosis. J Immunol (2010) 184(11):6275–82. doi: 10.4049/jimmunol.1000304 PMC2874741 20421645

[134] Lachmandas E Van Den Heuvel CNAM Damen MSMA Cleophas MCP Netea MG Van Crevel R. Diabetes Mellitus and Increased Tuberculosis Susceptibility: The Role of Short-Chain Fatty Acids. J Diabetes Res (2016) 2016:36–8. doi: 10.1155/2016/6014631 PMC4709651 27057552

[135] Lopez-Lopez N Martinez AGR Garcia-Hernandez MH Hernandez-Pando R Castañeda-Delgado JE Lugo-Villarino G. Type-2 Diabetes Alters the Basal Phenotype of Human Macrophages and Diminishes Their Capacity to Respond, Internalise, and Control Mycobacterium Tuberculosis. Mem Inst Oswaldo Cruz (2018) 113(4):1–11. doi: 10.1590/0074-02760170326 29513874 PMC5851047

[136] Restrepo BI Khan A Singh VK de-Leon E Aguillón-Durán GP Ledezma-Campos E. Human Monocyte-Derived Macrophage Responses to M. Tuberculosis Differ by the Host’s Tuberculosis, Diabetes or Obesity Status, and are Enhanced by Rapamycin. Tuberculosis (2021) 126:1–8. doi: 10.1016/j.tube.2020.102047 PMC7887072 33418150

[137] Torres M Herrera MT Fabián-San-Miguel G Gonzalez Y. The Intracellular Growth of M. Tuberculosis is More Associated With High Glucose Levels Than With Impaired Responses of Monocytes From T2D Patients. J Immunol Res (2019) 2019:1–10. doi: 10.1155/2019/1462098 PMC6877949 31815150

[138] Wang CH Yu CT Lin HC Liu CY Kuo HP. Hypodense Alveolar Macrophages in Patients With Diabetes Mellitus and Active Pulmonary Tuberculosis. Tuber Lung Dis (1999) 79(4):235–42. doi: 10.1054/tuld.1998.0167 10692992

[139] Martinez N Ketheesan N West K Vallerskog T Kornfeld H. Impaired Recognition of Mycobacterium Tuberculosis by Alveolar Macrophages From Diabetic Mice. J Infect Dis (2016) 214(11):1629–37. doi: 10.1093/infdis/jiw436 PMC5144731 27630197

[140] Hodgson K Morris J Bridson T Govan B Rush C Ketheesan N. Immunological Mechanisms Contributing to the Double Burden of Diabetes and Intracellular Bacterial Infections. Immunology (2015) 144(2):171–85. doi: 10.1111/imm.12394 PMC4298412 25262977

[141] Ferlita S Yegiazaryan A Noori N Lal G Nguyen T To K. Type 2 Diabetes Mellitus and Altered Immune System Leading to Susceptibility to Pathogens, Especially Mycobacterium Tuberculosis. J Clin Med (2019) 8(12):2219. doi: 10.3390/jcm8122219 PMC6947370 31888124

[142] Segura-Cerda CA López-Romero W Flores-Valdez MA. Changes in Host Response to Mycobacterium Tuberculosis Infection Associated With Type 2 Diabetes: Beyond Hyperglycemia. Front Cell Infect Microbiol (2019) 9:1–10. doi: 10.3389/fcimb.2019.00342 31637222 PMC6787561

[143] Palanisamy GS Kirk NM Ackart DF Obregón-Henao A Shanley CA Orme IM. Uptake and Accumulation of Oxidized Low-Density Lipoprotein During Mycobacterium Tuberculosis Infection in Guinea Pigs. PloS One (2012) 7(3):1–10. doi: 10.1371/journal.pone.0034148 PMC3320102 22493658

[144] Vrieling F Wilson L Rensen PCN Walzl G Ottenhoff THM Joosten SA. Oxidized Low-Density Lipoprotein (Oxldl) Supports Mycobacterium Tuberculosis Survival in Macrophages by Inducing Lysosomal Dysfunction. PloS Pathog (2019) 15(4):1–27. doi: 10.1371/journal.ppat.1007724 PMC6490946 30998773

[145] Alves C Casqueiro J Casqueiro J. Infections in Patients With Diabetes Mellitus: A Review of Pathogenesis. Indian J Endocrinol Metab (2012) 16(7):27. doi: 10.4103/2230-8210.94253 PMC3354930 22701840

[146] van Crevel R van de Vijver S Moore DAJ. The Global Diabetes Epidemic: What Does It Mean for Infectious Diseases in Tropical Countries? Lancet Diabetes Endocrinol (2017) 5(6):457–68. doi: 10.1016/S2213-8587(16)30081-X PMC7104099 27499355

[147] Nigam P Dayal SG Srivastava P Joshi LD Goyal BM Dutt B. Diabetic Status in Leprosy. Hansenol Int (1979) 4(1):7–14. 261975

[148] Okoye AA Picker LJ. Cd4+ T-Cell Depletion in Hiv Infection: Mechanisms of Immunological Failure. Immunol Rev (2013) 254(1):54–64. doi: 10.1111/imr.12066 23772614 PMC3729334

[149] Caruso AM Serbina N Klein E Triebold K Bloom BR Flynn JL. Mice Deficient in CD4 T Cells Have Only Transiently Diminished Levels of IFN-Gamma, Yet Succumb to Tuberculosis. J Immunol (1999) 162(9):5407–16. 10228018

[150] Scanga CA Mohan VP Yu K Joseph H Tanaka K Chan J. Depletion of CD4+ T Cells Causes Reactivation of Murine Persistent Tuberculosis Despite Continued Expression of Interferon γ and Nitric Oxide Synthase 2. J Exp Med (2000) 192(3):347–58. doi: 10.1084/jem.192.3.347 PMC2193220 10934223

[151] Lawn SD Myer L Edwards D Bekker LG Wood R. Short-Term and Long-Term Risk of Tuberculosis Associated With CD4 Cell Recovery During Antiretroviral Therapy in South Africa. Aids (2009) 23(13):1717–25. doi: 10.1097/QAD.0b013e32832d3b6d PMC3801095 19461502

[152] Wahl SM Allen JB Gartner S Orenstein JM Popovic M Chenoweth DE. HIV-1 and its Envelope Glycoprotein Down-Regulate Chemotactic Ligand Receptors and Chemotactic Function of Peripheral Blood Monocytes. J Immunol (1989) 142(10):3553–9. 2541200

[153] Spear GT Kessler HA Rothberg L Phair J Landay AL. Decreased Oxidative Burst Activity of Monocytes From Asymptomatic HIV-Infected Individuals. Clin Immunol Immunopathol (1990) 54(2):184–91. doi: 10.1016/0090-1229(90)90080-A 1688521

[154] Nicol MQ Mathys J-M Pereira A Ollington K Ieong MH Skolnik PR. Human Immunodeficiency Virus Infection Alters Tumor Necrosis Factor Alpha Production via Toll-Like Receptor-Dependent Pathways in Alveolar Macrophages and U1 Cells. J Virol (2008) 82(16):7790–8. doi: 10.1128/JVI.00362-08 PMC2519548 18524817

[155] Richardson ET Shukla S Sweet DR Wearsch PA Tsichlis PN Henry Boom W. Toll-Like Receptor 2-Dependent Extracellular Signal-Regulated Kinase Signaling in Mycobacterium Tuberculosis-Infected Macrophages Drives Anti-Inflammatory Responses and Inhibits Th1 Polarization of Responding T Cells. Infect Immun (2015) 83(6):2242–54. doi: 10.1128/IAI.00135-15 PMC4432743 25776754

[156] Porcheray F Léone C Samah B Rimaniol AC Dereuddre-Bosquet N Gras G. Glutamate Metabolism in HIV-Infected Macrophages: Implications for the CNS. Am J Physiol - Cell Physiol (2006) 291(4):618–26. doi: 10.1152/ajpcell.00021.2006 16687472

[157] Castellano P Prevedel L Valdebenito S Eugenin EA. HIV Infection and Latency Induce a Unique Metabolic Signature in Human Macrophages. Sci Rep (2019) 9(1):1–14. doi: 10.1038/s41598-019-39898-5 30850623 PMC6408492

[158] Hollenbaugh JA Munger J Kim B. Metabolite Profiles of Human Immunodeficiency Virus Infected CD4+ T Cells and Macrophages Using LC-MS/MS Analysis. Virology (2011) 415(2):153–9. doi: 10.1016/j.virol.2011.04.007 PMC3107887 21565377

[159] Bowman ER Cameron CM Richardson B Kulkarni M Gabriel J Cichon MJ. Macrophage Maturation From Blood Monocytes is Altered in People With HIV, and is Linked to Serum Lipid Profiles and Activation Indices: A Model for Studying Atherogenic Mechanisms. PloS Pathog (2020) 16(10):1–24. doi: 10.1371/journal.ppat.1008869 PMC7553323 33002093

[160] Bryson BD Rosebrock TR Tafesse FG Itoh CY Nibasumba A Babunovic GH. Heterogeneous GM-CSF Signaling in Macrophages is Associated With Control of Mycobacterium Tuberculosis. Nat Commun (2019) 10(1):1–11. doi: 10.1038/s41467-019-10065-8 31133636 PMC6536549

[161] Kumawat K Pathak SK Spetz AL Kundu M Basu J. Exogenous Nef is an Inhibitor of Mycobacterium Tuberculosis-Induced Tumor Necrosis Factor-α Production and Macrophage Apoptosis. J Biol Chem (2010) 285(17):12629–37. doi: 10.1074/jbc.M109.073320 PMC2857058 20068037

[162] Patel NR Zhu J Tachado SD Zhang J Wan Z Saukkonen J. Hiv Impairs Tnf-α Mediated Macrophage Apoptotic Response to Mycobacterium Tuberculosis. J Immunol (2007) 179(10):6973–80. doi: 10.4049/jimmunol.179.10.6973 17982088

[163] Patel NR Swan K Li X Tachado SD Koziel H. Impaired M. Tuberculosis -Mediated Apoptosis in Alveolar Macrophages From HIV+ Persons: Potential Role of IL-10 and BCL-3. J Leukoc Biol (2009) 86(1):53–60. doi: 10.1189/jlb.0908574 19383626 PMC2704623

[164] Lawn SD Pisell TL Hirsch CS Wu M Butera ST Toossi Z. Anatomically Compartmentalized Human Immunodeficiency Virus Replication in HLA-DR+ Cells and CD14+ Macrophages at the Site of Pleural Tuberculosis Coinfection. J Infect Dis (2001) 184(9):1127–33. doi: 10.1086/323649 11598835

[165] Schiff AE Linder AH Luhembo SN Banning S Deymier MJ Diefenbach TJ. T Cell-Tropic HIV Efficiently Infects Alveolar Macrophages Through Contact With Infected CD4+ T Cells. Sci Rep (2021) 11(1):1–14. doi: 10.1038/s41598-021-82066-x 33594125 PMC7886866

[166] Cribbs SK Lennox J Caliendo AM Brown LA Guidot DM. Healthy HIV-1-Infected Individuals on Highly Active Antiretroviral Therapy Harbor HIV-1 in Their Alveolar Macrophages. AIDS Res Hum Retroviruses (2015) 31(1):64–70. doi: 10.1089/aid.2014.0133 25134819 PMC4287110

[167] Jambo KC Banda DH Kankwatira AM Sukumar N Allain TJ Heyderman RS. Small Alveolar Macrophages are Infected Preferentially by HIV and Exhibit Impaired Phagocytic Function. Mucosal Immunol (2014) 7(5):1116–26. doi: 10.1038/mi.2013.127 PMC4009066 24472847

[168] Mazzolini J Herit F Bouchet J Benmerah A Benichou S Niedergang F. Inhibition of Phagocytosis in HIV-1-Infected Macrophages Relies on Nef-Dependent Alteration of Focal Delivery of Recycling Compartments. Blood (2010) 115(21):4226–36. doi: 10.1182/blood-2009-12-259473 20299515

[169] Deretic V Vergne I Chua J Master S Singh SB Fazio JA. Endosomal Membrane Traffic: Convergence Point Targeted by Mycobacterium Tuberculosis and HIV. Cell Microbiol (2004) 6(11):999–1009. doi: 10.1111/j.1462-5822.2004.00449.x 15469429

[170] Mwandumba HC Russell DG Nyirenda MH Anderson J White SA Molyneux ME. Mycobacterium Tuberculosis Resides in Nonacidified Vacuoles in Endocytically Competent Alveolar Macrophages From Patients With Tuberculosis and HIV Infection. J Immunol (2004) 172(7):4592–8. doi: 10.4049/jimmunol.172.7.4592 15034077

[171] Ustianowski AP Lawn SD Lockwood DN. Interactions Between HIV Infection and Leprosy: A Paradox. Lancet Infect Dis (2006) 6(6):350–60. doi: 10.1016/S1473-3099(06)70493-5 16728321

[172] Menezes VM Nery JAC Sales AM Miranda A Galhardo MCG Bastos FI. Epidemiological and Clinical Patterns of 92 Patients Co-Infected With HIV and Mycobacterium Leprae From Rio De Janeiro State, Brazil. Trans R Soc Trop Med Hyg (2014) 108(2):63–70. doi: 10.1093/trstmh/trt113 24361943

[173] da Silva TP Bittencourt TL de Oliveira AL Prata RB da S Menezes V Ferreira H. Macrophage Polarization in Leprosy–HIV Co-Infected Patients. Front Immunol (2020) 11:1–11. doi: 10.3389/fimmu.2020.01493 32849508 PMC7403476

[174] O’Brien DP Comte E Serafini M Ehounou G Antierens A Vuagnat H. The Urgent Need for Clinical, Diagnostic, and Operational Research for Management of Buruli Ulcer in Africa. Lancet Infect Dis (2014) 14(5):435–40. doi: 10.1016/S1473-3099(13)70201-9 24309480

[175] Vincent QB Ardant MF Marsollier L Chauty A Alcaïs A. HIV Infection and Buruli Ulcer in Africa. Lancet Infect Dis (2014) 14(9):796–7. doi: 10.1016/S1473-3099(14)70882-5 25164193

[176] Kiran D Podell BK Chambers M Basaraba RJ. Host-Directed Therapy Targeting the Mycobacterium Tuberculosis Granuloma: A Review. Semin Immunopathol (2016) 38(2):167–83. doi: 10.1007/s00281-015-0537-x PMC4779125 26510950

[177] Hawn TR Shah JA Kalman D. New Tricks for Old Dogs: Countering Antibiotic Resistance in Tuberculosis With Host-Directed Therapeutics. Immunol Rev (2015) 264(1):344–62. doi: 10.1111/imr.12255 PMC4571192 25703571

[178] Wallis RS Hafner R. Advancing Host-Directed Therapy for Tuberculosis. Nat Rev Immunol (2015) 15(4):255–63. doi: 10.1038/nri3813 25765201

[179] Chai Q Zhang Y Liu CH. Mycobacterium Tuberculosis: An Adaptable Pathogen Associated With Multiple Human Diseases. Front Cell Infect Microbiol (2018) 8:1–15. doi: 10.3389/fcimb.2018.00158 29868514 PMC5962710

[180] Naicker N Sigal A Naidoo K. Metformin as Host-Directed Therapy for TB Treatment: Scoping Review. Front Microbiol (2020) 11:1–11. doi: 10.3389/fmicb.2020.00435 32411100 PMC7201016

[181] Magee MJ Salindri AD Kornfeld H Singhal A. Reduced Prevalence of Latent Tuberculosis Infection in Diabetes Patients Using Metformin and Statins. Eur Respir J (2019) 53(3):1–4. doi: 10.1183/13993003.01695-2018 PMC6709848 30523163

[182] Singhal A Jie L Kumar P Hong GS Leow MKS Paleja B. Metformin as Adjunct Antituberculosis Therapy. Sci Transl Med (2014) 6(263):1–10. doi: 10.1126/scitranslmed.3009885 25411472

[183] Pan SW Yen YF Kou YR Chuang PH Su VYF Feng JY. The Risk of TB in Patients With Type 2 Diabetes Initiating Metformin vs Sulfonylurea Treatment. Chest (2018) 153(6):1347–57. doi: 10.1016/j.chest.2017.11.040 29253553

[184] Lin SY Tu HP Lu PL Chen TC Wang WH Chong IW. Metformin is Associated With a Lower Risk of Active Tuberculosis in Patients With Type 2 Diabetes. Respirology (2018) 23(11):1063–73. doi: 10.1111/resp.13338 29943489

[185] Degner NR Wang JY Golub JE Karakousis PC. Metformin Use Reverses the Increased Mortality Associated With Diabetes Mellitus During Tuberculosis Treatment. Clin Infect Dis (2018) 66(2):198–205. doi: 10.1093/cid/cix819 29325084 PMC5848303

[186] Lee YJ Han SK Park JH Lee JK Kim DK Chung HS. The Effect of Metformin on Culture Conversion in Tuberculosis Patients With Diabetes Mellitus. Korean J Intern Med (2018) 33(5):933–40. doi: 10.3904/kjim.2017.249 PMC6129638 29540054

[187] Łabuzek K Liber S Gabryel B Adamczyk J Okopień B. Metformin Increases Phagocytosis and Acidifies Lysosomal/Endosomal Compartments in AMPK-Dependent Manner in Rat Primary Microglia. Naunyn Schmiedebergs Arch Pharmacol (2010) 381(2):171–86. doi: 10.1007/s00210-009-0477-x 20012266

[188] Li X Fang P Mai J Choi ET Wang H Yang XF. Targeting Mitochondrial Reactive Oxygen Species as Novel Therapy for Inflammatory Diseases and Cancers. J Hematol Oncol (2013) 6(1):1–19. doi: 10.1186/1756-8722-6-19 23442817 PMC3599349

[189] Böhme J Martinez N Li S Lee A Marzuki M Tizazu AM. Metformin Enhances Anti-Mycobacterial Responses by Educating CD8+ T-Cell Immunometabolic Circuits. Nat Commun (2020) 11(1):1–15. doi: 10.1038/s41467-020-19095-z 33067434 PMC7567856

[190] Pellegrini JM Tateosian NL Morelli MP Rollandelli A Amiano NO Palmero D. Immunosuppressive Role of PGE2 During Human Tuberculosis Instituto De Química Biológica De La Facultad De Ciencias Exactas Y Naturales ( IQUIBICEN ). Universidad de Buenos Instituto de Histología y Embriología de: Facultad de Ciencias Exactas y Naturales (2020). Cp 5500.

[191] Moreno JR García IE de la Luz García Hernández M Leon DA Marquez R Pando RH. The Role of Prostaglandin E2 in the Immunopathogenesis of Experimental Pulmonary Tuberculosis. Immunology (2002) 106(2):257–66. doi: 10.1046/j.1365-2567.2002.01403.x PMC1782721 12047755

[192] Duan H Liu T Zhang X Yu A Cao Y. Statin Use and Risk of Tuberculosis: A Systemic Review of Observational Studies. Int J Infect Dis (2020) 93:168–74. doi: 10.1016/j.ijid.2020.01.036 31982626

[193] Hennessy E Adams C Reen FJ O’Gara F. Is There Potential for Repurposing Statins as Novel Antimicrobials? Antimicrob Agents Chemother (2016) 60(9):5111–21. doi: 10.1128/AAC.00192-16 PMC4997871 27324773

[194] Dutta NK Bruiners N Pinn ML Zimmerman MD Prideaux B Dartois V. Statin Adjunctive Therapy Shortens the Duration of TB Treatment in Mice. J Antimicrob Chemother (2016) 71(6):1570–7. doi: 10.1093/jac/dkw014 PMC5007636 26903278

[195] Parihar SP Guler R Khutlang R Lang DM Hurdayal R Mhlanga MM. Statin Therapy Reduces the Mycobacterium Tuberculosis Burden in Human Macrophages and in Mice by Enhancing Autophagy and Phagosome Maturation. J Infect Dis (2014) 209(5):754–63. doi: 10.1093/infdis/jit550 24133190

[196] Coleman MM Basdeo SA Coleman AM Cheallaigh CN De Castro CP McLaughlin AM. All-Trans Retinoic Acid Augments Autophagy During Intracellular Bacterial Infection. Am J Respir Cell Mol Biol (2018) 59(5):548–56. doi: 10.1165/rcmb.2017-0382OC 29852080

[197] Wheelwright M Kim EW Inkeles MS De Leon A Pellegrini M Krutzik SR. All- Trans Retinoic Acid–Triggered Antimicrobial Activity Against Mycobacterium Tuberculosis is Dependent on NPC2. J Immunol (2014) 192(5):2280–90. doi: 10.4049/jimmunol.1301686 PMC3954114 24501203

[198] Dürr UHN Sudheendra US Ramamoorthy A. LL-37, the Only Human Member of the Cathelicidin Family of Antimicrobial Peptides. Biochim Biophys Acta - Biomembr (2006) 1758(9):1408–25. doi: 10.1016/j.bbamem.2006.03.030 16716248

[199] Liu PT Stenger S Tang DH Modlin RL. Cutting Edge: Vitamin D-Mediated Human Antimicrobial Activity Against Mycobacterium Tuberculosis Is Dependent on the Induction of Cathelicidin. J Immunol (2007) 179(4):2060–3. doi: 10.4049/jimmunol.179.4.2060 17675463

[200] Martineau AR Timms PM Bothamley GH Hanifa Y Islam K Claxton AP. High-Dose Vitamin D3 During Intensive-Phase Antimicrobial Treatment of Pulmonary Tuberculosis: A Double-Blind Randomised Controlled Trial. Lancet (2011) 377(9761):242–50. doi: 10.1016/S0140-6736(10)61889-2 PMC4176755 21215445

[201] Coussens AK Wilkinson RJ Hanifa Y Nikolayevskyy V Elkington PT Islam K. Vitamin D Accelerates Resolution of Inflammatory Responses During Tuberculosis Treatment. Proc Natl Acad Sci USA (2012) 109(38):15449–54. doi: 10.1073/pnas.1200072109 PMC3458393 22949664

[202] Borah K Beyß M Theorell A Wu H Basu P Mendum TA. Intracellular Mycobacterium Tuberculosis Exploits Multiple Host Nitrogen Sources During Growth in Human Macrophages. Cell Rep (2019) 29(11):3580–3591.e4. doi: 10.1016/j.celrep.2019.11.037 31825837 PMC6915324

[203] Koeken VACM Lachmandas E Riza A Matzaraki V Li Y Kumar V. Role of Glutamine Metabolism in Host Defense Against Mycobacterium Tuberculosis Infection. J Infect Dis (2019) 219(10):1662–70. doi: 10.1093/infdis/jiy709 30541099

[204] Rapovy SM Zhao J Bricker RL Schmidt SM Setchell KDR Qualls JE. Differential Requirements for L-Citrulline and L-Arginine During Antimycobacterial Macrophage Activity. J Immunol (2015) 195(7):3293–300. doi: 10.4049/jimmunol.1500800 PMC6432794 26311904

[205] El Kasmi KC Qualls JE Pesce JT Smith AM Thompson RW Henao-Tamayo M. Toll-Like Receptor-Induced Arginase 1 in Macrophages Thwarts Effective Immunity Against Intracellular Pathogens. Nat Immunol (2008) 9(12):1399–406. doi: 10.1038/ni.1671 PMC2584974 18978793

[206] Lange SM McKell MC Schmidt SM Zhao J Crowther RR Green LC. L-Arginine Synthesis From L-Citrulline in Myeloid Cells Drives Host Defense Against Mycobacteria In Vivo. J Immunol (2019) 202(6):1747–54. doi: 10.4049/jimmunol.1801569 PMC6401247 30710047

[207] Mattila JT Ojo OO Kepka-Lenhart D Marino S Kim JH Eum SY. Microenvironments in Tuberculous Granulomas are Delineated by Distinct Populations of Macrophage Subsets and Expression of Nitric Oxide Synthase and Arginase Isoforms. J Immunol (2013) 191(2):773–84. doi: 10.4049/jimmunol.1300113 PMC3746594 23749634

[208] Zhang YJ Reddy MC Ioerger TR Rothchild AC Dartois V Schuster BM. Tryptophan Biosynthesis Protects Mycobacteria From CD4 T-Cell-Mediated Killing. Cell (2013) 155(6):1296–308. doi: 10.1016/j.cell.2013.10.045 PMC3902092 24315099

[209] Abate Negatu D Yamada Y Xi Y Lin Go M Zimmerman M Ganapathy U. Gut Microbiota Metabolite Indole Propionic Acid Targets Tryptophan Biosynthesis in Mycobacterium Tuberculosis. MBio (2019) 10(2):1–15. doi: 10.1128/mBio.02781-18 PMC6437058 30914514

[210] Blumenthal A Nagalingam G Huch JH Walker L Guillemin GJ Smythe GA. M. Tuberculosis Induces Potent Activation of Ido-1, But This is Not Essential for the Immunological Control of Infection. PloS One (2012) 7(5):1–11. doi: 10.1371/journal.pone.0037314 PMC3359358 22649518

[211] Mehra S Pahar B Dutta NK Conerly CN Philippi-Falkenstein K Alvarez X. Transcriptional Reprogramming in Nonhuman Primate (Rhesus Macaque) Tuberculosis Granulomas. PloS One (2010) 5(8):1–12. doi: 10.1371/journal.pone.0012266 PMC2930844 20824205

[212] Moura-Alves P Faé K Houthuys E Dorhoi A Kreuchwig A Furkert J. Ahr Sensing of Bacterial Pigments Regulates Antibacterial Defence. Nature (2014) 512(7515):387–92. doi: 10.1038/nature13684 25119038

[213] Kim JS Kim YR Yang CS. Host-Directed Therapy in Tuberculosis: Targeting Host Metabolism. Front Immunol (2020) 11:1–12. doi: 10.3389/fimmu.2020.01790 32903583 PMC7438556

[214] Park JH Shim D Kim KES Lee W Shin SJ. Understanding Metabolic Regulation Between Host and Pathogens: New Opportunities for the Development of Improved Therapeutic Strategies Against Mycobacterium Tuberculosis Infection. Front Cell Infect Microbiol (2021) 11:1–21. doi: 10.3389/fcimb.2021.635335 PMC8007978 33796480

[215] Crowther RR Qualls JE. Metabolic Regulation of Immune Responses to Mycobacterium Tuberculosis: A Spotlight on L-Arginine and L-Tryptophan Metabolism. Front Immunol (2021) 11:1–16. doi: 10.3389/fimmu.2020.628432 PMC7900187 33633745

[216] Kiran D Basaraba RJ. Lactate Metabolism and Signaling in Tuberculosis and Cancer: A Comparative Review. Front Cell Infect Microbiol (2021) 11(37):624607. doi: 10.3389/fcimb.2021.624607 33718271 PMC7952876

[217] Lobato LS Rosa PS Da Silva Ferreira J Da Silva Neumann A Da Silva MG Nascimento DC. Statins Increase Rifampin Mycobactericidal Effect. Antimicrob Agents Chemother (2014) 58(10):5766–74. doi: 10.1128/AAC.01826-13 PMC4187984 25049257

[218] Castillo EF Dekonenko A Arko-Mensah J Mandell MA Dupont N Jiang S. Autophagy Protects Against Active Tuberculosis by Suppressing Bacterial Burden and Inflammation. Proc Natl Acad Sci USA (2012) 109(46):1–9. doi: 10.1073/pnas.1210500109 PMC3503152 23093667

[219] Saini NK Baena A Ng TW Venkataswamy MM Kennedy SC Kunnath-Velayudhan S. Suppression of Autophagy and Antigen Presentation by Mycobacterium Tuberculosis PE-PGRS47. Nat Microbiol (2016) 1(52):1–12. doi: 10.1038/nmicrobiol.2016.133 PMC5662936 27562263

[220] Evans MJ Levy L. Ultrastructural Changes in Cells of the Mouse Footpad Infected With Mycobacterium Leprae. Infect Immun (1972) 5(2):238–47. doi: 10.1128/iai.5.2.238-247.1972 PMC422354 4564400

[221] Silva BJ de A Barbosa MG de M Andrade PR Ferreira H Nery JA da C Côrte-Real S. Autophagy Is an Innate Mechanism Associated With Leprosy Polarization. PloS Pathog (2017) 13(1):1–29. doi: 10.1371/journal.ppat.1006103 PMC5215777 28056107

[222] Sieling PA Modlin RL. Cytokine Patterns at the Site of Mycobacterial Infection. Immunobiology (1994) 4–5:378–87. doi: 10.1016/S0171-2985(11)80443-2 7713551

[223] Dang AT Teles RMB Weiss DI Parvatiyar K Sarno EN Ochoa MT. IL-26 Contributes to Host Defense Against Intracellular Bacteria. J Clin Invest (2019) 129(5):1926–39. doi: 10.1172/JCI99550 PMC6486355 30939123

